# Generalizability and transferability of machine learning models using hyperspectral reflectance data for maize traits

**DOI:** 10.1038/s41598-026-36819-1

**Published:** 2026-01-21

**Authors:** Rudan Xu, John Ferguson, Matthieu Breil-Aubert, Johannes Kromdijk, Zoran Nikoloski

**Affiliations:** 1 Bioinformatics Department, Institute of Biochemistry and Biology, University of Potsdam, Potsdam, Germany; 2 Systems Biology and Mathematical Modelling Group, Max Planck Institute of Molecular Plant Physiology, Potsdam, Germany; 3 School of Life Sciences, University of Essex, Colchester, UK; 4 Department of Plant Sciences, University of Cambridge, Cambridge, UK

**Keywords:** Hyperspectral reflectance, Machine learning, Zea mays, Model generalizability, Anatomical traits, Chlorophyll fluorescence, Gas exchange., Computational biology and bioinformatics, Plant sciences

## Abstract

Hyperspectral reflectance provides rapid, non-destructive phenotyping of plant leaves. These data have been used to develop machine learning models for predicting diverse plant traits, yet key challenges remain. We collected hyperspectral reflectance data together with 25 anatomical, gas exchange, and chlorophyll fluorescence traits from 320 recombinant inbred lines grown over three seasons. Using these data, we systematically (1) compare the performance of PLSR and SVR across a wide range of traits, including also slow fluorescence kinetics, (2) assess model generalizability and transferability, and (3) investigate how different aggregation strategies affect predictive accuracy. Based on a nested cross-validation framework, single cross-validation with MSE as metric performed comparably to repeated cross-validation or PRESS-based calibration. Optimal performance of trait-specific predictions was found to be dependent on the combination of model and data aggregation levels. Structural and biochemical traits showed the best generalizability and transferability, whereas physiological traits, particularly those derived from gas exchange and fluorescence kinetics, exhibited markedly reduced transferability. Together, these results provide a rigorous benchmark for evaluating machine learning models for trait prediction from hyperspectral reflectance data, and highlight both the opportunities and limitations for achieving robust generalization across diverse environments and genotypes.

**Supplementary Information:**

The online version contains supplementary material available at 10.1038/s41598-026-36819-1.

## Introduction

Efforts to develop climate-resilient crops go hand-in-hand with advances in high-throughput phenotyping technologies that can be deployed at scale in accelerated breeding^1,2^. Urgent innovations in high-throughput profiling are needed given that many breeding targets involve molecular and biochemical traits that are difficult to measure across large breeding populations^3^. Hyperspectral reflectance (HSR) offers a promising solution to this problem by enabling rapid, precise, and non-destructive phenotyping in both controlled and field environments^4^. HSR captures reflected light across a continuous range of wavelengths spanning the visible (400–700 nm), near-infrared (700–1300 nm), and short-wavelength infrared (1400–3000 nm) regions. The resulting spectral provide detailed fingerprints of leaf structural and biochemical properties, such as pigment composition, water content and nitrogen status, thereby revealing underlying physiological processes^5^. For example, nitrogen-containing chemical bonds modify spectral features associated with leaf nitrogen content^6^, whilst reflectance at the red to near-infrared transition, particularly near the first spectral peak, decreases with increasing Rubisco-limited carboxylation rate (V_cmax_)^7^.

Leveraging these relationships, numerous studies have used machine learning (ML) models to predict a wide range of biochemical and physiological traits from HSR across diverse plants species^8–19^. Using these ML models, different traits, such as: Vcmax, the electron transport-limited rate (J_max_), the triose phosphate utilization-limited rate, and leaf nitrogen content per area, and specific leaf area (SLA), have been predicted with high accuracy^20,21^. Despite these successes, several methodological aspects of HSR-based ML modeling remain underexplored. Most studies have relied on linear models, e.g., partial least squares regression (PLSR)^9–14,17,19^ or linear kernel support vector regression (SVR)^22,23^, whilst the usage of non-linear models including random forest^19^, gradient boosting^23,24^ and deep learning methods^15,16,18^ is less common. However, model calibration procedures, particularly the tuning of the number of components (NoC) for PLSR models, vary substantially between studies, and their impact in model validation performance has not been systematically evaluated.

A second major gap concerns model generalizability and transferability. Generalizability refers to prediction performance on data drawn from the same distribution as the training set (e.g., random cross-validation splits involving genotypes, species and environments present during training), whereas transferability captures the ability to extrapolate to new distributions, including unseen genotypes, species, seasons, or their combination. While many studies assessed model generalizability using cross validation or permutation-based validation based on random data split, only a few explicitly examined whether models can transfer well to new genotypes and species, or to distinct growing conditions such as greenhouse versus field environments or different field seasons^11,12^. Even in studies that include multi-season datasets or combined greenhouse and field measurement^12,13,17,22^, model transferability has been rarely evaluated in a rigorous fashion.

**Fig. 1:**
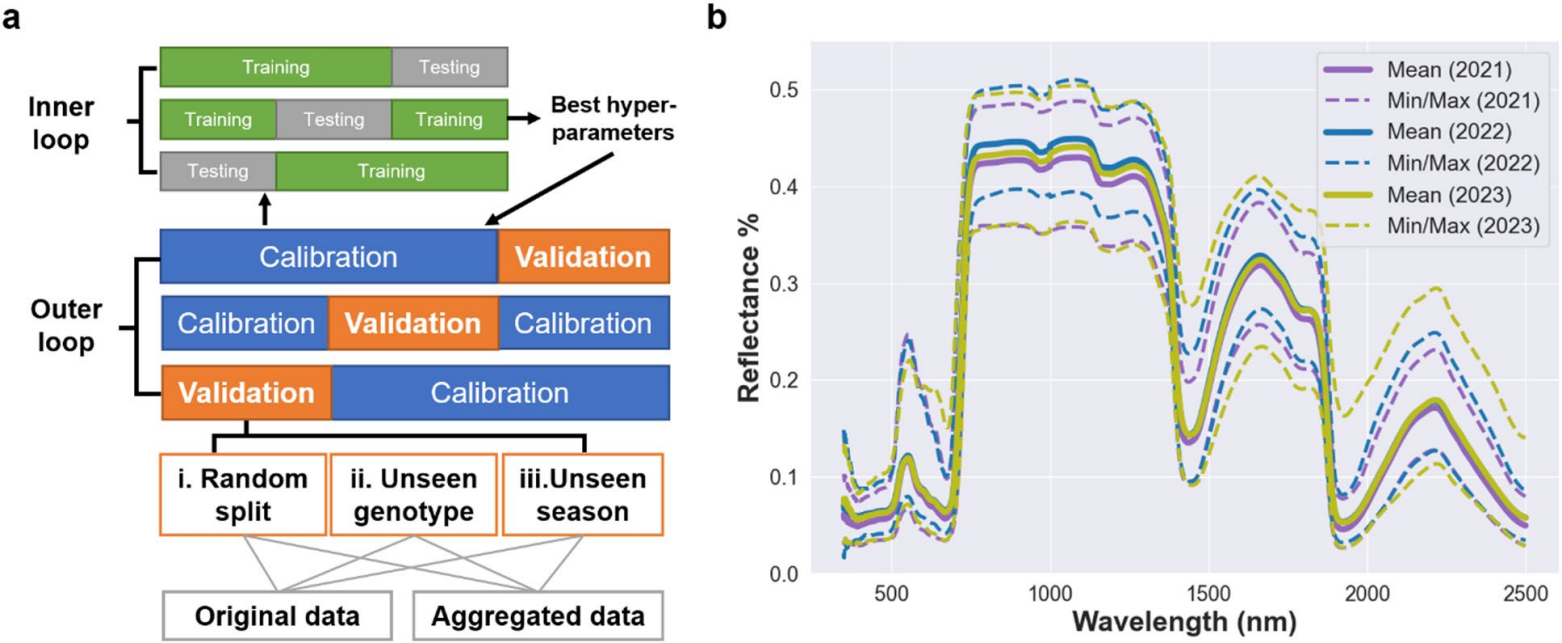
Illustration of the machine learning framework for prediction of physiological traits using HSR data. (a) Scheme of the repeated nested CV procedure. The outer-loop partitions the data into a calibration set (blue) and a validation set (orange) in each iteration. The validation set is used exclusively to assess the model generalizability under three prediction scenarios: (i) random unseen samples, (ii) unseen genotypes, and (iii) unseen seasons. Within each calibration set, the inner-loop further divides the data into on training (green) and testing (gray) subsets to optimize model hyperparameters. The best hyperparameter values derived from the inner loop are then applied to the outer-loop calibration and validation sets. Both the original and aggregated data were evaluated under this framework. (b) Summary of HSR data measured on 320 lines from a maize MAGIC population grown in field experiments in three consecutive seasons (2021–2023). Solid lines represent the mean HSR values over the genotypes in each season, while dashed lines denote the corresponding minimum and maximum reflectance values across genotypes.

Here, we collected HSR data and paired measurements of 25 traits from 320 recombinant inbred lines (RILs) of a maize Multi-Parent Advanced Generation Inter-Cross (MAGIC) population grown across three consecutive seasons. Using this data set, we addressed the following key questions:

1. How can one select the optimal NoC in PLSR in an accurate and computationally efficient way? To address this question, we systematically compared different inner-loop structures and selection criteria using repeated cross-validation and permutation-based approach to assess their impact on model performance and uncertainty.
2. How do PLSR and SVR models perform under increasingly stringent scenarios of generalization? To this end, we evaluated three levels of model generalization: (i) random splits of training and validation data; (ii) prediction of unseen genotypes within the same season; and (iii) prediction of traits from unseen seasons (Fig. 1a). We note that these scenarios of model generalizability are particularly relevant for making decisions based on the ML models in breeding.
3. How do data aggregation and season influence model performance? In this respect, we investigated the impact of averaging raw trait and reflectance measurements at the plot or genotype level. In addition, we investigated how aggregation affects the model generalization scenarios, mentioned above. To this end, we made use of the three replicates of the genotypes per plot, to compare models that are based on HSR and traits data aggregated by averaging over plots or averaging over genotypes.

By addressing these questions, our study provides a robust experimental and analytical framework for assessing model performance on HSR data from field trials, with direct implications for predictive phenotyping across genotypes, traits, and environments.

## Results

### Phenotyping a maize MAGIC population gathers large-scale data suitable for testing the generalizability of ML models

To address our questions, we gathered leaf HSR data obtained from a maize MAGIC population grown in three consecutive field seasons (i.e., 2021, 2022 and 2023) (Fig. 1b, Methods). The average HSR profiles from three seasons over all samples were consistent across the measured wavelength region of 700 to 3500 nm. In the visible region (350–700 nm), both the minimum and maximum HSR values over all samples were similar across the seasons. However, by calculating the correlation of averaged HSR per genotype, the mean correlation based on the data from this region ranged from 0.27 to 0.36 between three seasons (Table S1). In the near-infrared region (700–1300 nm), where HSR reaches the high plateau, the minimum values measured in 2022 were significantly higher than those measured in 2021 and 2023, while the maximum values in 2021 were lower than those in 2022 and 2023 (Fig. 1b). The average correlation for this region across genotypes between seasons 2022 and 2023 was 0.5, which was lower than the correlation of 0.57 observed between the 2021 and 2022 seasons and the 0.53 observed between the 2021 and 2023 season. The shortwave infrared region (1300–2500 nm) also showed differences in the range of HSR values across the three seasons, with averaged correlation of 0.50, 0.44 and 0.49 for the pairs of three seasons (Table S1). Moreover, Mantel tests were performed to assess if the covariance matrix of HSR data (across genotypes) in one season were correlated with the covariance matrix from another season. We found that seasons 2021 and 2022 showed a correlation of 0.89, seasons 2021 and 2023 had a correlation of 0.88 and seasons 2022 and 2023 showed a correlation of 0.93. These values indicated differences in the covariance structure of the HSR data that may affect generalizability and transferability of the models, particularly involving training / testing with data from season 2021 that differs the most from data from 2022 to 2023.

In addition, 25 traits were measured or estimated during these seasons, with their distribution depicted in Figs. S1–S3. These traits were grouped into three categories, with different number of samples in each category (Table S2). We measured six anatomical traits, including: specific leaf area, relative carbon content (%C), relative nitrogen content (%N), carbon to nitrogen ratio (C: N) as well as the stable carbon and nitrogen isotope ratios (δ^13^C and δ^15^N). Seven gas exchange traits were estimated from the Farquhar-von Caemmerer-Berry (FvCB) model (Von Caemmerer, 2000) using *A-Ci* response curves, namely: stomatal limitation, photosynthetic rate, stomatal conductance, and intrinsic water use efficiency (iWUE) at saturating light, maximum rate of carboxylation by phosphoenolpyruvate carboxylase (V_pmax_), asymptote of the *A-Ci* curve (V_max_). Moreover, we considered 13 chlorophyll fluorescence traits, related to photosystem II (PSII) operating efficiency (ΦPSII), PSII quantum efficiency (Fv/Fm) and response of non-photochemical quenching (NPQ).

We observed that δ^15^N, stomatal limitation, and NPQ induction rate exhibited the highest average coefficient of variation (CVs) within season (CVs > 45%), while δ^13^C, Fv/Fm and final ΦPSII showed CVs below 5%. We also observed that V_pmax_ had higher variability compared to V_max_, with average CVs of 39% and 24%, respectively. To assess the consistency of the measurements across seasons, we determined the Spearman correlation between seasons for each trait, averaged per genotype (Table S3). We found that, %C, δ^15^N and iWUE, on average, had the lowest correlation between seasons, with mean correlation values of 0.02, 0.21 and 0.24, respectively. In contrast, fluorescence traits such as NPQ relaxation amplitude, ΦPSII recovery offset and ΦPSII induction amplitude showed the highest correlation between seasons, with values of 0.60, 0.62 and 0.64. Interestingly, SLA and C: N had correlation of 0.43 and 0.53 while V_pmax_ and V_max_ averaged correlations of 0.32 and 0.47. These results suggested variability in both HSR and traits data between seasons, indicating presence of genotype-by-environmental interaction, that may limit the transferability of models between seasons.

### PLSR model sensitivity to the NoC estimation

In this section, we first compared two strategies for NoC estimation: repeated CV partitioning with MSE as performance metric^26,34^ and sub-sampling with PRESS statistics^25^. For the first approach a fixed design of 20 repetitions of 5-fold CV was implemented and the optimal NoC, ${\mathrm{No}}{{\mathrm{C}}_{{\mathrm{MSE}}\left( {{\mathrm{opt}}} \right)}}$, was selected for each repetition, while the most frequently chosen value across 20 repetitions was defined as the final estimated NoC: $No{C_{MSE}}$^26^ (Fig. S4). The distribution of ${\mathrm{No}}{{\mathrm{C}}_{{\mathrm{MSE}}\left( {{\mathrm{opt}}} \right)}}$ values thus capture variability of NoC selection under different data partitions and allows assessment of its impact on model performance. For the second approach, 100 random 80/20 training-testing sub-sampling was employed to calculate PRESS statistic^30^ and select the optimal NoC (Fig. S4). For this analysis, traits measured in the 2021 season with their paired HSR ranged from 400 to 2400 nm were used. Exceptions were made for %N and C/N, for which only the 1500–2400 nm range was used, similar to previous studies^10,27^.

Overall, NoC estimates were largely consistent between these two strategies (Tables S4 and S5). Difference between $No{C_{MSE}}$ and $No{C_{PRESS}}$ were observed in twelve traits, with the largest discrepancies (∆=4) seen for SLA, NPQ induction slope, and Fv/Fm. Median R^2^ differed by less than 0.03 across all traits, suggesting strong agreement between the two selection strategies. Notably the PRESS-based method tended to favor slightly smaller NoC values for some traits.

**Fig. 2:**
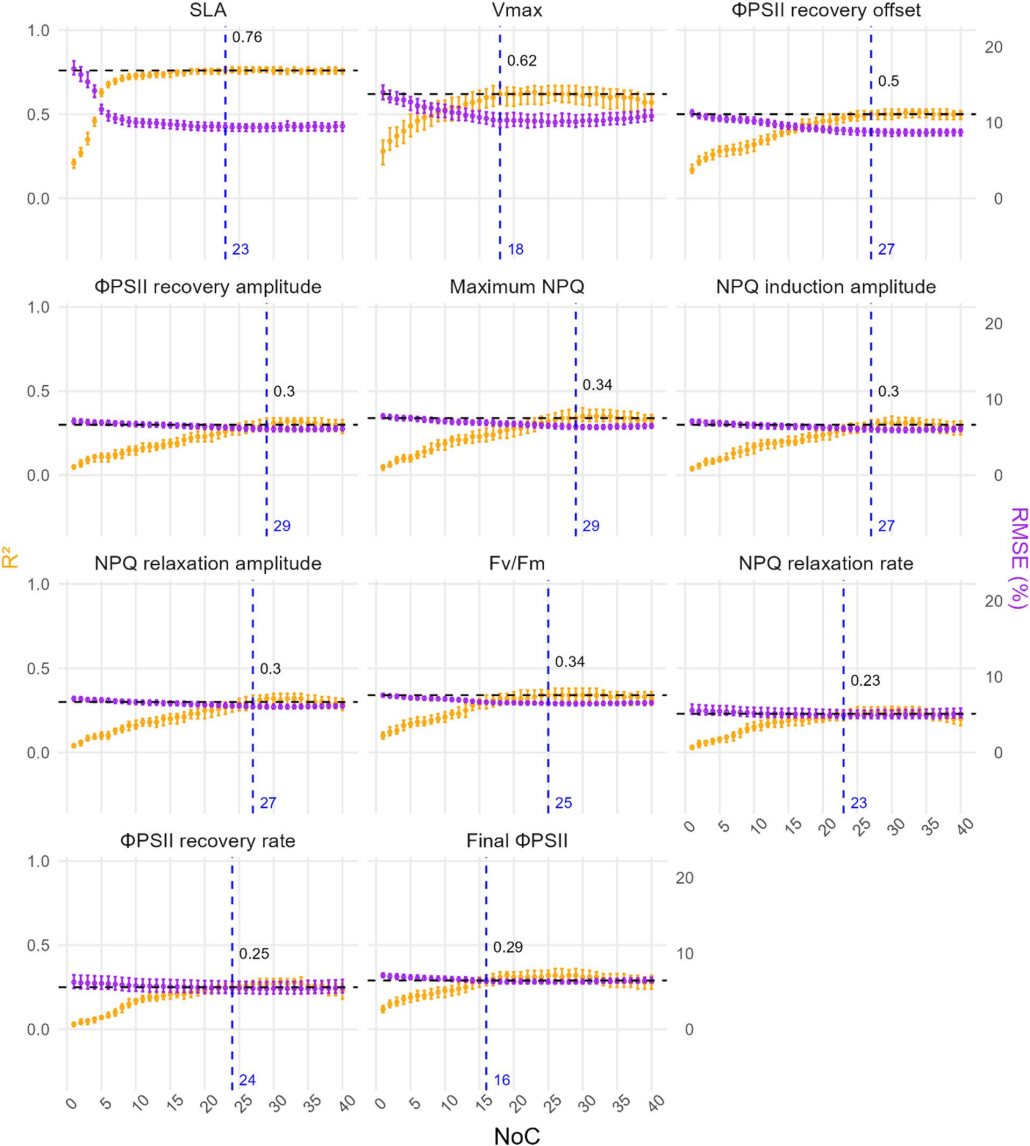
Sensitivity of PLSR model calibration to the number of component (NoC) across eleven selected traits measured in season 2021. Each panel shows model performances as a function of NoC values. These selected traits showed stable and robust performance across NoC. Model performance was evaluated using 20 repetitions of 5-fold cross-validation across varying NoC values. The coefficient of determination (R^2^, orange, left y-axis) and RMSE% (purple, right y-axis) are shown in dots as median values across the 100 test folds, with error bars representing the interquartile range (25th to 75th percentile). Vertical dashed lines denote the optimal number of components, $No{C_{MSE}}$, while horizontal dashed lines denote the corresponding median R^2^.

Most traits exhibited stable NoC estimation across the 20 repetitions, with the standard deviation (SD) of ${\mathrm{No}}{{\mathrm{C}}_{{\mathrm{MSE}}\left( {{\mathrm{opt}}} \right)}}$ ranging from 0 to 3 (22 traits; Table S4). δ^13^C and stomatal conductance (gsw) showed slightly higher variability (SD = 4), with corresponding median R^2^ values of 0.12 and 0.23 respectively. Notably, δ^15^N and NPQ induction rate showed the highest variability (SD = 7) and had near-zero median R^2^, suggesting a lack of predictive signal in the HSR data. We compared model calibration performance using the consensus $No{C_{MSE}}$ and individual ${\mathrm{No}}{{\mathrm{C}}_{{\mathrm{MSE}}\left( {{\mathrm{opt}}} \right)}}$ (Table S4). Twelve traits showed slightly higher median R^2^ when using ${\mathrm{No}}{{\mathrm{C}}_{{\mathrm{MSE}}\left( {{\mathrm{opt}}} \right)}}$, five traits performed better with $No{C_{MSE}}$, and the remaining traits showed no difference in R^2^. The standard deviations of R^2^ under both strategies were nearly identical, suggesting that most of the performance variability across iterations originated from differences in data partitioning rather than variation in ${\mathrm{No}}{{\mathrm{C}}_{{\mathrm{MSE}}\left( {{\mathrm{opt}}} \right)}}$. This can be explained that even the optimal NoC varied slightly across repetitions, such variability had small effect on overall model calibration performance, which generally plateaued within a stable NoC range (Fig. 2).

Next, we explore the sensitivity of PLSR calibration performance to increasing NoC values, ranging from 1 to 40, by evaluating the root mean squared error percentage (RMSE%) and coefficient of determination (R^2^) under the framework repeated CV partition. Based on the shape of the R^2^ response to increasing NoC across the repeated CV, the 25 traits were grouped into four categories:

1. Traits with stable and robust performance across NoC (Fig. 2). These traits exhibited increasing R^2^ and decreasing RMSE% with increasing NoC, followed by a performance plateau within the selected range, where model accuracy stabilized and variability across CV splits was small (Table S4). SLA showed the highest calibration accuracy (median R^2^ = 0.76 with $No{C_{MSE}}$=23). Other traits with moderate predictability included V_max_ (median R^2^ = 0.62, $No{C_{MSE}}$=18) and several chlorophyll fluorescence traits such as ΦPSII recovery offset, ΦPSII induction amplitude, maximum NPQ, NPQ induction amplitude, NPQ relaxation amplitude and Fv/Fm, which achieved median R^2^ between 0.30 and 0.50, with estimated $No{C_{MSE}}$ exceeding 20. While some traits like NPQ relaxation rate, final ΦPSII and ΦPSII induction rate showed a median R^2^ ranged between 0.11 and 0.29. These traits maintained robust model performance even at high NoC values, with small variation across repetitions.
2. Traits with moderate predictability but overfitting beyond optimal NoC (Fig. S5). For this group, R^2^ improved initially but declined beyond the optimal NoC region. Examples include %N, C/N, δ^13^C, *A* and gsw, all of which showed relatively higher variability across CV folds. Their median R^2^ at $No{C_{MSE}}$ ranged from 0.12 to 0.56, suggesting that the model captured a meaningful predictive signal, despite the eventual decline in performance due to overfitting at higher levels of model complexity.
3. Traits with low predictability and overfitting beyond optimal NoC (Fig. S5). Traits such as %C, δ^15^N, V_pmax_, Stomatal limitation, iWUE and NPQ induction slope exhibited low R^2^ (< 0.15) at low NoC and decreasing R^2^ at higher NoC, with large variability across CV folds. These patterns suggested weak predictive signal and poor generalizability.
4. Traits with flat and low R^2^ curves across NoC values (Fig. S5). For traits such as NPQ induction rate, NPQ relaxation offset and final NPQ, R^2^ remained close to zero across the entire NoC range, with minimal variation across different folds, indicating that the model was unable to capture any meaningful relationship between the predictors and the response variable. Consequently, predictions were limited to the mean response, and increasing model complexity did not lead to performance deterioration.

To examine the influence of spectral resolution, we compared models trained on the full HSR data set (2001 wavelengths from 400 nm to 2400) with models trained on a sub-sampled data set retaining every fifth wavelength (401 wavelengths). The resulting median R^2^ and optimal NoC estimation were highly similar between the two approaches, indicating that wavelength sub-sampling offers an efficient form of dimension reduction without compromising predictive accuracy (Table S6). Consequently, the sub-sampled HSR data were used for all downstream analyses.

### Generalizability of PLSR and SVR using different data aggregation strategies

To assess within-season generalizability of PLSR, we implemented a 5-fold CV repeated 20 times as the outer loop (Fig. 1), following Filzmoser et al.^26^. Because calibration performance did not differ markedly between $No{C_{MSE}}$ and individual ${\mathrm{No}}{{\mathrm{C}}_{{\mathrm{MSE}}\left( {{\mathrm{opt}}} \right)}}$ values, we compared their impact on validation accuracy. Two inner-loop strategies were therefore used: 10 times repeated 3-fold CV to estimate the consensus $No{C_{MSE}}$, and a single CV iteration to determine ${\mathrm{No}}{{\mathrm{C}}_{{\mathrm{MSE}}\left( {{\mathrm{opt}}} \right)}}$ for each outer loop iteration. As expected, ${\mathrm{No}}{{\mathrm{C}}_{{\mathrm{MSE}}\left( {{\mathrm{opt}}} \right)}}$ varied more across iterations than $No{C_{MSE}}$, but this variability did not significantly impact the final validation R^2^ (Fig. S6). Strong predictive performance (median R^2^ > 0.5) was achieved for SLA, %N, C:N ratio, and V_max_. Moderate performance (0.3 < median R^2^ < 0.5) was observed for *A*, NPQ induction and relaxation amplitudes, ΦPSII recovery amplitude and offset, final ΦPSII, Fv/Fm, and maximum NPQ. The remaining thirteen traits exhibited median R^2^ lower than 0.3 across three seasons and were therefore not illustrated and were not considered for downstream analyses. Compared to repeated CV calibration, we observed that single-CV calibration was faster and yielded comparable validation performance, and it was used in nested CV framework in the subsequent analyses.

The 2021 data set included up to six HSR-trait measurement pairs per recombinant inbred line (RIL), derived from two replications (plot) per line arranged in an alpha-lattice design, with three replicates in each plot. These raw data were aggregated either at the plot or at the genotype level. Using PLSR with sub-sampled HSR data, we evaluated how data aggregation strategies affected model performance. Genotype-averaged data improved model accuracy for structural and biochemical traits such as SLA, %N, and C: N ratio, with median R^2^ increasing from 0.75, 0.50, and 0.56 (raw) to 0.81, 0.74, and 0.74, respectively (Fig. S7). While *A* performed better using raw data (median R^2^ = 0.36), V_max_ showed improvement from plot-level averaging (median R^2^ = 0.59). Most fluorescence traits performed best with raw data, whereas ΦPSII recovery offset, final ΦPSII and Fv/Fm showed improved predictive accuracy when using plot-averaged data, with median R^2^ increasing from 0.49, 0.30 and 0.32 to 0.5, 0.38 and 0.42, respectively. Aggregation strategy also influenced NoC estimation: genotype averaging generally reduced the estimated NoC.

Data from three consecutive seasons allowed testing whether these effects of aggregation were consistent using PLSR. SLA consistently showed the greatest improvement with genotype-averaged data across all seasons (Fig. S8). Due to the absence of raw biochemical traits measurements for 2022 and 2023, only plot- and genotype-level aggregations could be evaluated. For %N and C: N ratio, genotype-level averaging led to the most substantial improvements across all seasons, increasing median R^2^ values from 0.64 to 0.68 and 0.71–0.73 (with plot-level data) to 0.72–0.74 and 0.78–0.79 (genotype level), respectively. In contrast, most photosynthesis and chlorophyll fluorescence traits performed best using raw measurements, suggesting that fine-scale within-plot variability contained relevant spectral signal that was lost during aggregation. Exceptions again included ΦPSII recovery offset, final ΦPSII and Fv/Fm, which consistently showed improved predictability with plot-averaged data across all seasons.

These patterns are consistent with the variance structure revealed by linear mixed-effects models. For structural and biochemical traits, genotype-level averaging substantially reduced residual variance, reflecting lower within-genotype heterogeneity after aggregation (Table S7). Conversely, for most physiological traits, residual variance increased with genotype-level averages, suggesting that aggregation removed informative leaf-level variation and amplified unexplained variability, in line with their superior prediction at the raw measurement level.

**Fig. 3:**
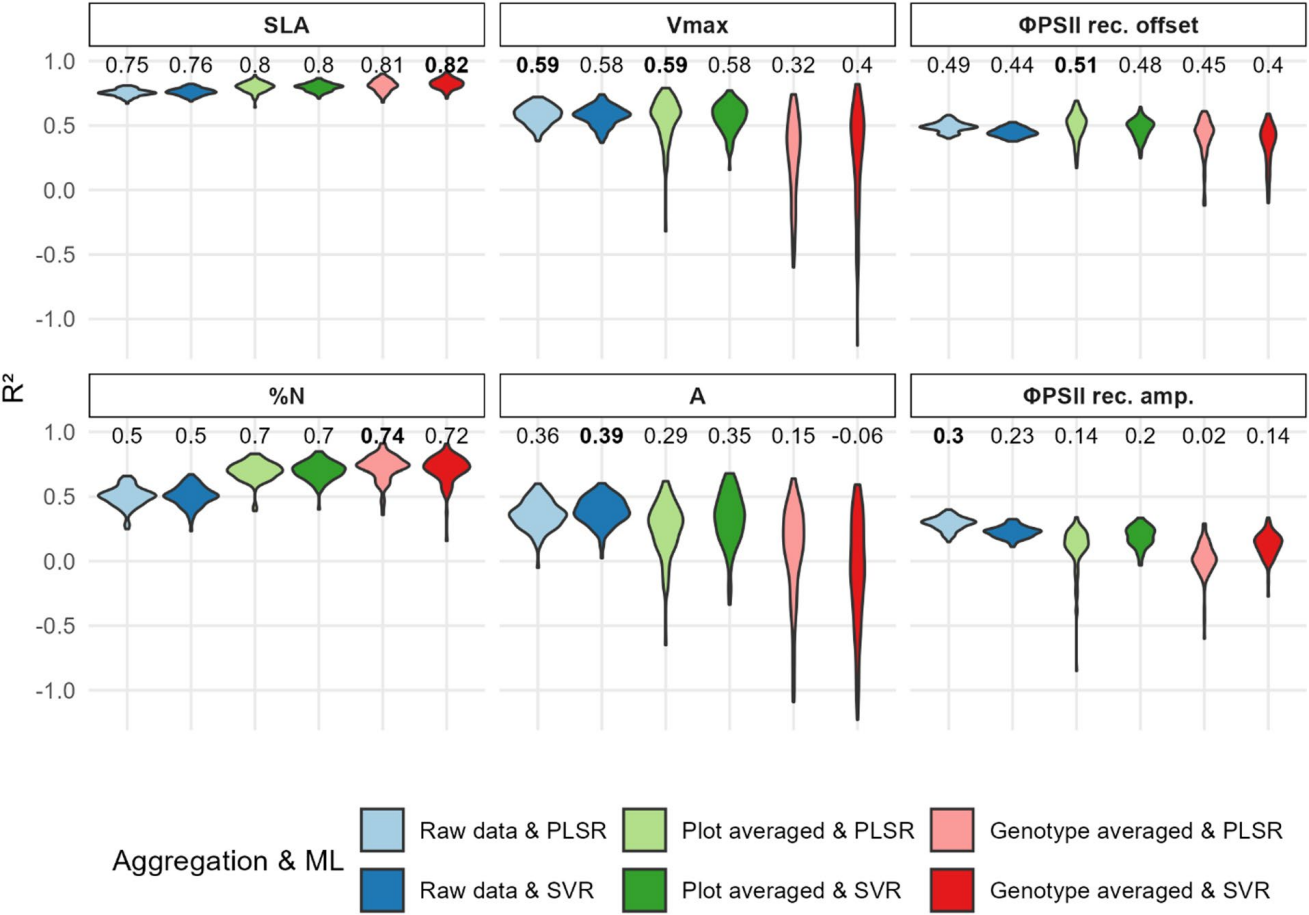
Comparison of PLSR and SVR model performance under different aggregation strategies for six selected traits measured in season 2021. The top row displays the best-performing traits from each of the three categories, namely structural traits, traits extracted from gas exchange and from chlorophyll fluorescence. The bottom row displays traits with relatively lower predictive performance. Each trait was evaluated using combinations of aggregation methods and machine learning algorithms (color-coded), with model performance assessed via 20 repetitions of 5-fold CV, calibrated with single CV. Violin plots depict the distribution of R^2^ scores across 100 validation folds, with median values shown above each plot. Bolded numbers indicate the highest median R^2^ achieved among all combinations.

To benchmark our findings against another linear method, we applied linear-kernel SVR using the same outer-loop framework. SVR produced results comparable to PLSR with single-CV calibration and slightly outperformed it for traits such as SLA and *A* in season 2021 (Fig. 3). Aggregation effects across different traits were consistent for both ML methods (Figs. 3, S9), indicating that the improvements observed were driven by data structure rather than model-specific tuning. To further test whether SVR consistently outperform PLSR for certain traits across seasons, trait-specific aggregation strategies were employed: for SLA, %N and C: N ratio, genotype-averaged data were used; for ΦPSII recovery offset, final ΦPSII and Fv/Fm, plot-averaged data were used, while raw data was used for the remaining traits. SVR outperformed PLSR for SLA, *A* and V_max_ across all three seasons while for final ΦPSII, SVR had better performance for 2021 and 2023 (Fig. S10). Physiological traits also exhibited larger difference in predictability across seasons, with 2021 consistently showing the best performance.

The previous evaluations assessed model generalizability across genotypes within single seasons. To explore whether combining seasons improve performance and model robustness, we tested all two- and three-season combinations using the optimal model-aggregation pairing for each trait: genotype-averaged data with PLSR for SLA, %N, and C: N ratio; plot-averaged data with PLSR for ΦPSII recovery offset, final ΦPSII and Fv/Fm; and raw data with SVR for the remaining traits. While several traits, including C: N ratio, NPQ relaxation amplitude, ΦPSII recovery amplitude and offset, final ΦPSII, Fv/Fm and maximum NPQ, performed the best using data set from individual season, multi-season calibration slightly improved median R^2^ for the remaining traits (Fig. S11). Notably, multi-season calibration consistently reduced prediction uncertainty, reinforcing the advantage of integrating environmental variability to improve model robustness.

Together, these results underscore the importance of tailoring data aggregation strategies to the specific trait categories and compare machine learning models to optimize model performance and reliability across environmental conditions in different seasons.

### Evaluating model transferability to unseen genotypes and seasons

The analyses above utilized random split between calibration and validation sets, applied to individual seasons or combination of three seasons, enabling a systematic assessment of how data aggregation strategies and seasonal variation affect the predictive performance of PLSR and SVR models. To investigate model transferability, we assessed how well these models predict traits for genotype not present during calibration within a season. This analysis used sub-sampled HSR data and the same nested cross-validation framework as before, with single-CV calibration for NoC estimation and trait-specific combinations of ML methods and aggregation strategies. Importantly, genotypes were strictly at both CV levels: in the inner loop, training and testing folds contained no overlapping genotypes, and in the outer loop, calibration and validation folds were likewise genotype-exclusive. This prevented data leakage and ensured that models were evaluated only on unseen genotypes.

**Fig. 4:**
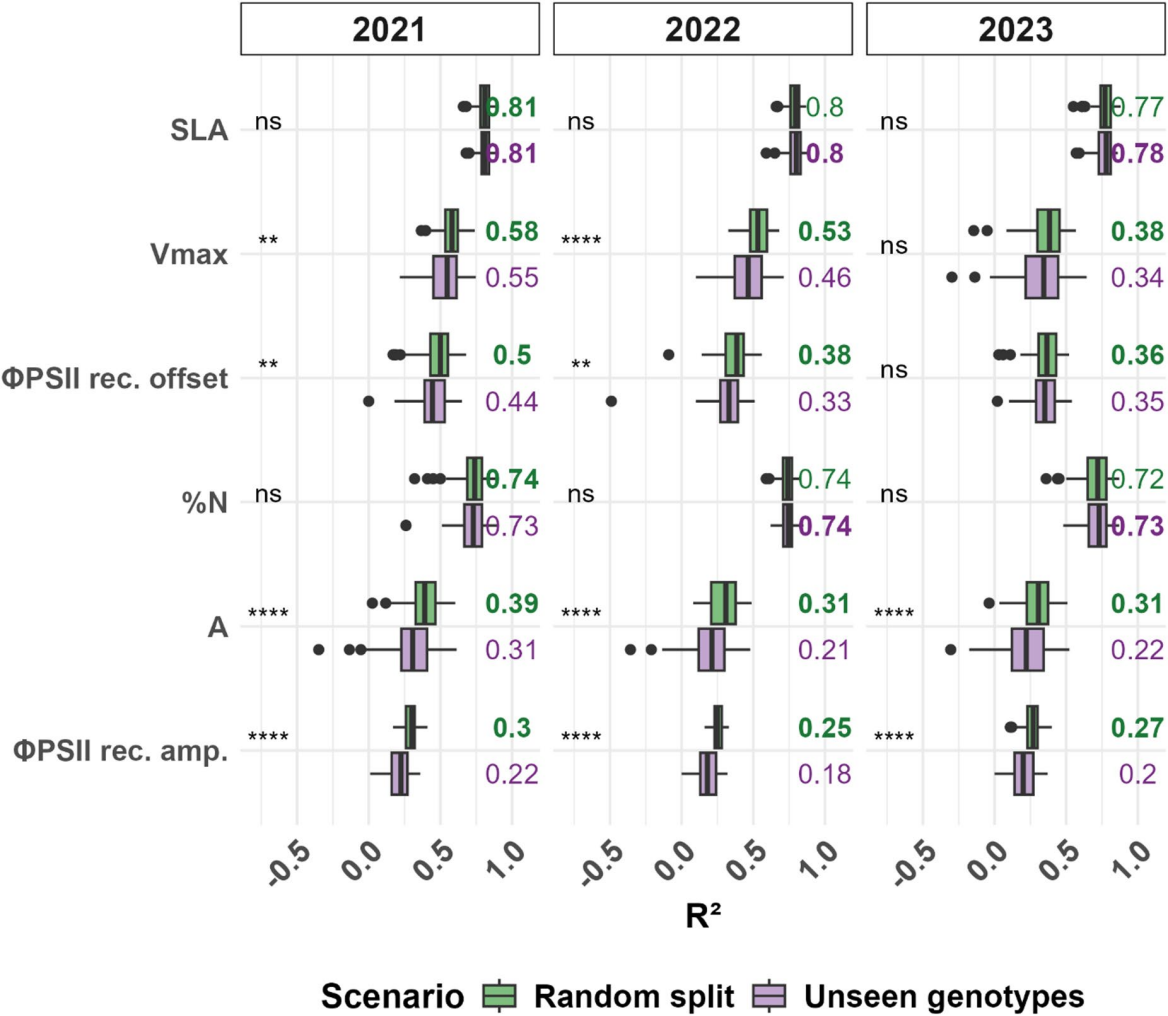
Comparison of prediction performance between random and unseen genotype scenarios using trait-specific combinations of aggregation strategies and machine learning algorithms. For selected six traits measured across three seasons, boxplots show coefficient of determination (R^2^) values across 20 repetitions of 5-fold CV, using the optimal combination of aggregation method and machine learning algorithm for each trait. Two prediction scenarios were evaluated: random data points splitting calibration and validation(green) and validation includes only unseen genotypes (purple). Wilcoxon test was applied with statistical significance between scenarios indicated by asterisks: * (*p* ≤ 0.05), ** (*p* ≤ 0.01), *** (*p* ≤ 0.001), **** (*p* ≤ 0.0001), ns = not significant.

Genotype-averaged SLA, %N and C: N ratio demonstrated the strongest generalizability, exhibiting no significant decline in R^2^ values when moving from predicting randomly split data to predicting unseen genotypes across all three seasons (Figs. 4, S12). Plot-averaged ΦPSII recovery offset, final ΦPSII and Fv/Fm showed a smaller performance drop between the two scenarios than other fluorescence traits, whereas gas exchange traits had a marked decrease in predictive accuracy when moving to the unseen genotype scenario.

We next evaluate model transferability to other seasons by training on one or more seasons and testing on a held-out season. For instance, when predicting 2021 data, models were trained on data from 2022, 2023, or their combination, calibrated using 10 times repeated 3-fold CV for NoC estimation. Given the differences in trait magnitudes between seasons, model performance was assessed using squared Pearson correlation between predicted and observed values.

**Fig. 5:**
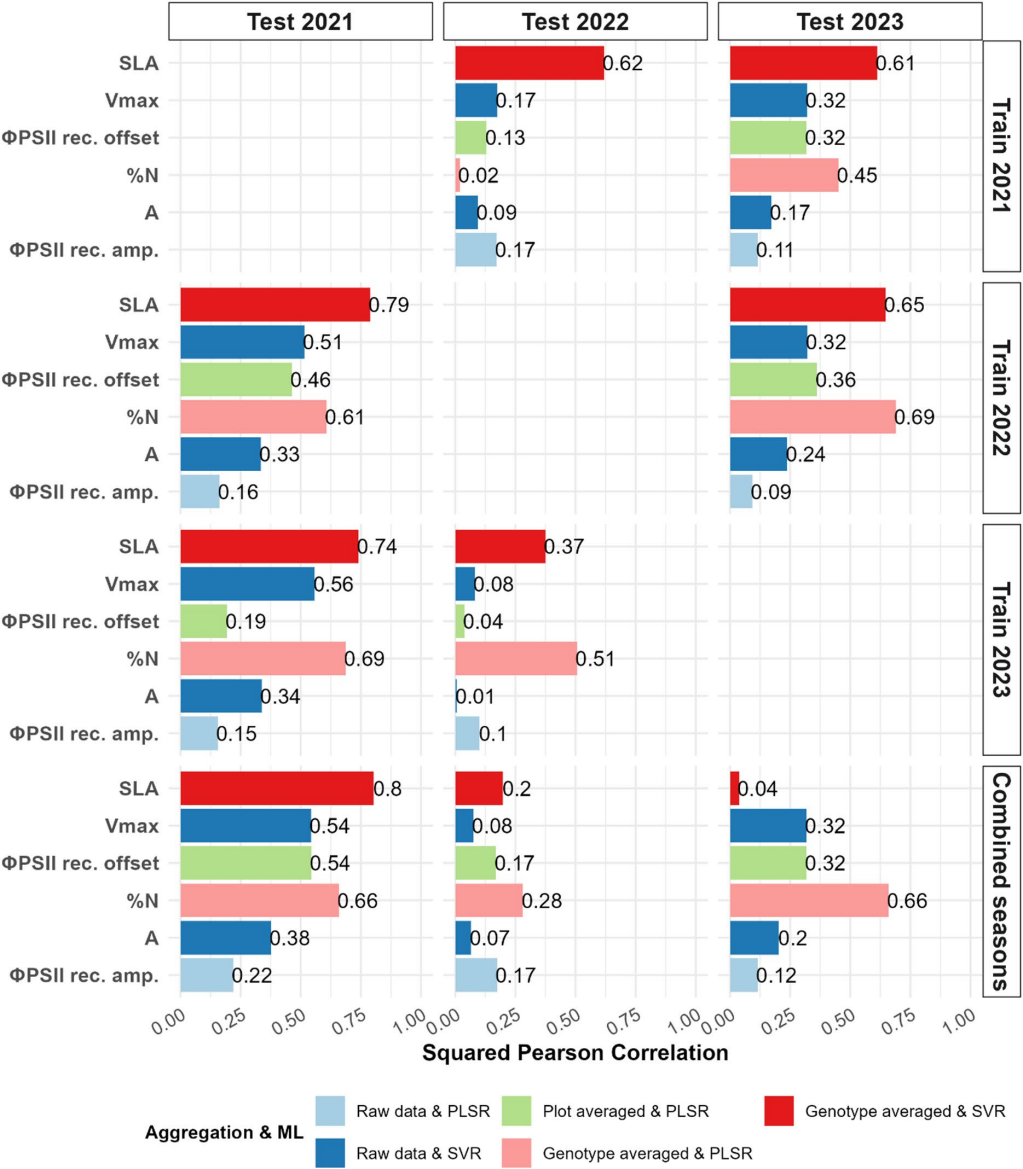
Prediction performance across unseen seasons using optimal aggregation and ML combinations. Bar plots display the squared Pearson correlation between predicted and measured trait values for selected traits. Rows indicate the season(s) used to calibrate the models (2021, 2022, 2023, or combined seasons), while columns correspond to the test season used for independent validation. For each trait, models were built using the trait-specific optimal combination of aggregation strategy and machine learning algorithm (illustrated in different colors).

Among the three tested seasons, season 2021 consistently showed the highest predictive accuracy, particularly when models were trained on combined data from 2022 to 2023 (Figs. 5, S13). In contrast, 2022 emerged as the most difficult season to predict, with limited gains based on combining data from 2021 to 2023. SLA, %N and C: N were the only traits for which models trained on individual seasons produced acceptable accuracy. When predicting 2023, training on the combined data sets from 2021 to 2022 resulted in the best overall performance across most traits, except SLA, for which models trained on individual seasons performed better. The poor performance of models trained on combined data from 2021 to 2022 (predicting 2023) and 2021–2023 (predicting 2022) for SLA was due to difference in SLA distributions in 2021 relative to the other two seasons (Fig. S1). Scaling and centering SLA within each season before model training corrected this mismatch and markedly improved squared correlations: from squared Pearson correlation of 0.20 to 0.60 for predicting season 2022 and from 0.04 to 0.6 for season 2023 (Fig. S14). However, scaling and centering had negligible effect on the remaining traits, as their distributions were largely similar across seasons.

Another challenge included the prediction of leaf nitrogen content (%N), which exhibited poor performance when transferring models from the 2021 season to 2022, despite successful prediction in the reverse direction (from 2022 to 2021). The 2022 dataset encompassed much broader spectral variability because it included 219 genotypes not measured in 2021, whereas the 2021 data covered only 99 genotypes that were all also present in 2022. This restricted genotype representation in 2021 resulted in a narrower range of spectral profiles (Fig. S15), which likely limited the ability of models trained on 2021 data to generalize to the more diverse 2022 population. This aligned with the findings of Ji et al.^33^, showing that higher spectral diversity in the training set improves PLSR generalizability in new domains. Similarly, predictions of V_max_ and *A* were declined markedly in season 2022 when models were trained on data from the other seasons. Examination of the HSR signatures revealed that, for accessions common across seasons, reflectance in 2022 exhibited a noticeably elevated plateau between 750 and 1500 nm (Fig. S16). In this region, most samples from 2021 fell within the broader 2022 reflectance distribution; however, a substantial portion of high-reflectance samples observed in 2022 were absent from the 2021 distribution, indicating a clear shift in spectral characteristics across seasons. Taken together, the limited number of genotypes with photosynthetic measurements and the pronounced differences in HSR distributions across seasons likely contributed to the reduced model transferability for these traits.

Beyond these distributional and spectral differences, variance component analysis further highlighted the importance of genotype-by-environment (GxE) interactions in shaping cross-season predictive performance (Table S7). Notably, gas-exchange traits exhibited the largest year variance, reflecting their strong environmental sensitivity, whereas chlorophyll fluorescence traits showed the largest accession variance, indicating stronger genetic control. Structural and biochemical traits (e.g., SLA, %N, C:N) generally showed low GxE variance relative to genotypic variance. This indicates that genotype rankings for these traits were largely stable across seasons, which is consistent with their relatively robust cross-season predictability. When mean temperature and photosynthetically active radiation (PAR) from the day prior to measurement were included as fixed effects in the linear mixed-effects models, the overall variance patterns remained largely unchanged (Table S8). This indicated that major seasonal differences were not driven by the variation in temperature and PAR.

Finally, we evaluated the most stringent scenario: predicting traits for unseen genotypes from entirely unseen seasons. For each target season, genotypes were partitioned into five folds for validation, while the remaining genotypes from the other two seasons were used for training. Genotype separation was enforced in both inner- and outer-loops and repeated for 20 times. The prediction performance evaluated squared Pearson correlations was significantly lower than in any previous scenario (Fig. S17). Particularly, the drop of predictive accuracy from predicting unseen within the same season to predicting unseen genotypes from unseen seasons (Fig. S17) was larger than the accuracy decline from random split to unseen genotypes prediction within the same season (Figs. 4, S12). Similar to the previous analysis, where the entire unseen season was predicted, here, to predict unseen genotypes from unseen season, 2022 was consistently the most challenging one.

Collectively, these results underscore the complexity of transferring hyperspectral models across genotypes and seasons. They highlight the importance of trait-specific properties, data aggregation strategies, and environmental variability in determining model robustness.

## Discussion

HSR data have been widely used to train ML models for predicting biochemical and physiological traits across diverse plant species and crops^8–14,16–19^. Despite this growing application, several key challenges remain insufficiently examined. First, the increasing variety of ML methods, each requiring appropriate hyperparameter tuning, raises questions regarding model choice and robustness. Second, the generalizability of the ML models within seasons and predicting seen genotypes, as well as their transferability to unseen genotypes and seasons has not been fully explored. Third, although multiple data aggregation strategies are possible in field-based studies, their impact on model performance has not been systematically evaluated.

To address these gaps, we evaluated model performance across increasingly realistic and challenging prediction scenarios: (1) predicting randomly split data within season(s), (2) prediction of unseen genotypes within a season, and (3) prediction of unseen seasons and of unseen genotypes from entirely unseen seasons. Using a multi-season field data set from a maize MAGIC population, we also assessed how model choice and data aggregation strategy influence predictive performance.

In the first part of our study, we calibrated PLSR models using the 2021 data set, evaluated model sensitivity to NoC, and compared three approaches for NoC estimation: repeated CV (MSE-based), single CV (MSE-based), and permutation-based selection using PRESS statistics. All three approaches yielded consistent NoC estimates and similar performance on testing folds, indicating that single-CV calibration is both sufficient and efficient for obtain reliable NoC values, particularly within a nested CV framework. Sub-sampling the spectra (retaining every fifth wavelength) yielded performance nearly identical to that of the full spectrum, while reducing computational time substantially. Optimal NoC for fluorescence traits are generally higher NoC (~ 30) reflecting that these traits involve multiple pigments, biochemical reactions and dynamic regulation mechanisms, which resulted in complex and distributed spectral signals. In contrast, most other traits showed an initial rise in accuracy followed by a plateau and subsequent decline at higher NoC values, with photosynthetic traits exhibiting greater variability across repetitions than structural and biochemical traits. Since the complete 2021 data set was used to tune NoC, this initial analysis served as a calibration step rather than an assessment of generalizability.

To rigorously evaluate model generalizability, we employed a nested CV design, consisting of 20 outer loops of 5-fold CV. For each repetition, the inner loop estimated NoC using either repeated CV or single CV with the MSE criterion, while the outer loop assessed model performance under different prediction scenarios. In the first scenario we evaluated within-season prediction of PLSR and SVR using random splits of raw, plot-averaged and genotype- averaged data across the 2021, 2022, and 2023 seasons.

Across all seasons and aggregation strategies, thirteen traits, including: %C, δ^13^C, δ^15^N, V_pmax_, gsw, iWUE, SL, NPQ induction rate and slope, NPQ relaxation rate and offset, final NPQ and ΦPSII recovery rate showed consistently low prediction accuracy (maximum median R^2^ < 0.3). The poor predictability of V_pmax_, estimated from the slope of the *A-Ci* curve under low internal CO_2_, likely reflects its dependence on more complex C_4_ photosynthetic mechanisms (C_4_ acid transport, CO_2_ leakiness, and energy partitioning), that may be difficult to capture by HSR alone^10^. NPQ induction traits also performed poorly, likely because NPQ induction represents a rapid, electrochemically driven process with weak structural signatures, whereas NPQ relaxation and ΦPSII recovery involve pigment transitions and structural dynamics more easily captured by HSR. Consistent with this, rate-based metrics (e.g., NPQ induction and relaxation rates as well as ΦPSII recovery rate) showed less predictive accuracy than amplitude-based metrics (e.g., NPQ relaxation amplitude, ΦPSII recovery amplitude), likely because amplitudes capture longer-term physiological changes detectable in reflectance, whereas rates reflect rapid processes upon light on/off switch with weak structural signatures^35^.

Among the remaining traits, structural and biochemical traits such as SLA, %N, and C: N ratio, demonstrated strong generalizability across all seasons and aggregation strategies. These traits benefited most from genotype-level averaging, which consistently improved prediction accuracy using both PLSR and SVR. Among photosynthetic traits, V_max_, representing the asymptotic maximum of the *A-Ci* curve under CO_2_-saturated Rubisco, demonstrated the highest generalizability, whereas *A*, measured at a fixed *Ci* of 400 ppm, was more affected by transient environmental variability, resulting in lower predictive accuracy. Both were found to be best predicted using raw data combined with linear SVR. Among fluorescence traits, ΦPSII recovery offset, reflecting the quantum efficiency of PSII at the end of light exposure, achieved the best performance with plot-averaged data; Similarly, Fv/Fm, representing the maximum PSII efficiency, and final ΦPSII showed moderate performance using plot-averaged data. For traits such as maximum NPQ, NPQ induction and relaxation amplitudes, and ΦPSII recovery amplitude, raw data outperformed other levels.

Importantly, no single ML method or aggregation strategy performed best across all traits; instead, optimal performance required trait-specific combinations of model choice and aggregation level. Although calibrating models using multiple seasons reduced prediction uncertainty in this scenario, improvements in accuracy were limited, especially for physiological traits.

In the second scenario, we examined model transferability to unsee genotypes grown in the same year as the calibration data. The decline in predictive performance compared to previous scenario with random split was not significant for SLA, %N, and C: N across seasons, while more significant difference was observed for photosynthetic traits.

When models were trained on one season and evaluated on a completely unseen season, prediction accuracy varied substantially across years, with the highest performance observed when predicting 2021 and the lowest when predicting 2022. Using data from two seasons for calibration slightly improved the accuracy compared to single-season calibration. We also found that difference in SLA distribution across seasons reduced prediction accuracy, an issue that could be addressed by centering and scaling SLA within each season. The most challenging setting involved predicting unseen genotypes from an entirely unseen season, which is also the most relevant scenario for breeding and remote-sensing applications. The substantial drop in performance in comparison to within-season scenarios highlighted the influence of environmental variation on the predictive power and transferability of HSR-based models.

Variance component analysis further supported these findings by revealing strong trait-dependent differences in genetic and environmental influences. Gas-exchange traits were dominated by year variance, reflecting their high environmental sensitivity, whereas fluorescence traits showed the largest variance due to genotype. Structural and biochemical traits exhibited relatively low GxE contributions, consistent with their more stable cross-season predictability. Importantly, adding temperature and PAR as covariates did not substantially change these patterns, suggesting that additional unmeasured environmental factors likely drive the reduced model transferability across seasons.

Our work paves the way for scrutinizing the predictive power of HSR data for different classes of traits by usage of well-established CV techniques and ML models. The calibration analysis showed that sub-sampled HSR data combined with single-CV calibration using the MSE criterion provides an efficient and reliable approach for NoC estimation under nested CV evaluation framework. In addition, it also points out that data transformations can have often drastic effect on model performance, particularly for prediction scenarios relevant in practice, e.g., breeding. By assessing model generalizability and transferability, we found that HSR is well suited for predicting integrative traits, like SLA and %N, while physiological traits, such as, ΦPSII recovery and maximum PSII efficiency, exhibited more moderate performance. Short-term and dynamic traits remain difficult to predict from HSR data alone, particularly when models are applied across seasons. These findings highlight the need for future work on advanced feature engineering and temporal modeling approaches to improve prediction of dynamic physiological processes.

## Materials and methods

### Leaf physiological traits and reflectance measurements

Field trials incorporating the maize MAGIC population^36^ were performed in 2021, 2022, and 2023 at the National Institute of Agricultural Botany (NIAB, Cambridge, UK). In each year, 320 recombinant inbred lines (RILs) were grown in a twice-replicated alpha lattice design. The experimental design has previously been described in full detail^35^. Some RILs were specific to certain years, but 305 RILs were grown and phenotyped in all three years.

All phenotyping was performed on the leaf subtending the ear, which was excised at dawn and returned to the laboratory for phenotyping, as described previously^35^. We have previously shown that this approach generated data comparable to measuring leaves still intact to the plant^37^.

Initially, light-saturated photosynthesis (*A*) was determined on all leaves using LI-6400 infra-red gas analyzers equipped with 6400-40 leaf chamber fluorometer LED light sources (LI-COR Inc., Lincoln, NE). Conditions within the leaf chambers were as follows: 1800 µmol m-2 s-1 photosynthetically active radiation (PAR), 25 °C block temperature, 400 µmol s-1 air flow, 65% relative humidity (RH), 400 µmol mol-1 reference CO_2_ concentration. A randomly selected subset of RILs (78 in 2021, 88 in 2022, 91 in 2023) were used for measuring the response of photosynthesis to changes in the intracellular concentration of CO_2_ (*A-Ci* curve) using LI-6800 infra-red gas analysers equipped with standard 6cm2 leaf chambers (LI-COR Inc., Lincoln, Nebraska). *A-Ci* response curves were collected exactly as described previously^38^. Using the *A-Ci* data, estimates of the maximum rate of carboxylation by phosphoenolpyruvate carboxylase (PEPC; V_pmax_) and the asymptote of the *A-Ci* curve (V_max_) were modeled following von Caemmerer^39^.

Leaf hyperspectral reflectance was measured on the same portion of the leaf where gas exchange was performed using an ASD FieldSpec 4 Standard-Res Spectroradiometer equipped with a leaf-clip (Malvern Panalytical, Malvern, UK). The light source of the FieldSpec was allowed to warm up for 45 min prior to measurements. For each leaf, three technical replicates were performed and the reflectance at each wavelength was averaged before subsequent data analyses.

Following hyperspectral reflectance, a section of leaf tissue (~ 2 × 4 cm) was excised from the previously measured leaf area for measurements of chlorophyll fluorescence to determine the quantum efficiency of photosystem II (Fv/Fm) as well as the response of non-photochemical quenching (NPQ) and photosystem II (PSII) operating efficiency (ΦPSII) to an actinic light being switched on and off. These measurements are as described previously^35^. Traits describing NPQ induction and relaxations, as well as ΦPSII recovery responses were obtained from exponential models. The 2021 and 2022 chlorophyll fluorescence data have previously been reported^35^.

### Data processing

For each genotype, measurements of different traits were collected from two plots, with three replicates each. To assess differences in model performance, raw data were averaged by plot or genotype. Wavelengths from 400 nm to 2400 nm were used in training models to predict each of the traits, except for nitrogen percentage and ratio of nitrogen to carbon percentage. For these traits, wavelengths from 1500 nm to 2400 nm were used, as in Yendrek et al.^10^. To account for the variability of trait response across seasons, particularly for SLA, before combining different seasons as training data, the trait values from each year were centered and scaled.

### Machine learning models

Supervised machine learning (ML) was used to develop models to predict measured traits based on HSR data. Once trained, these models can predict traits for which only reflectance data are available. Given that the traits are continuous variables, regression-based ML models were used. The predictions were performed by using ‘pls’^40^ and ‘caret’ package^41^, implemented in R software environment.

Partial least-squares regression (PLSR) is a state-of-art ML model commonly used in modeling plant traits based on HSR data as predictors^42,43^. HSR typically contain more predictors (i.e., reflectance at different wavelengths) than samples, and these predictors are often highly correlated, leading to a multicolinearity problem. PLSR addresses these challenges by projecting the response variable onto uncorrelated components, which are linear combinations of predictors. This approach allows the response to be expressed in terms of the original predictors by considering the response during component extraction. The number of components is a crucial hyperparameter in PLSR and is typically tuned using different procedures, as described above. PLSR has been successfully used to predict traits such as V_max_, J_max_ and %N across different species^10,21^.

For fairness of comparison to the widely used PLSR models based on HSR data, we considered linear kernel SVR to avoid nonlinearity of other kernels as explanation for differences in performance. As a result, the SVR also has one hyperparameter, *C*, referred to as the regularization hyperparameter. In this study, we implemented a nested CV workflow to tune the hyperparameter and validate the model using unseen data under different scenarios.

### Model calibration

PLSR is known to be highly sensitive to the number of components (NoC), which must be carefully selected during model calibration to balance predictive accuracy and overfitting risk. However, the criteria and procedures used for selecting the optimal NoC and for evaluating model performance vary considerably among studies. Two main approaches are commonly used to train and evaluate PLSR models- proposed by Burnett et al.^25^ and Filzmoser et al.^26^. The first approach, used, for instance, in Serbin et al.^9,27^, Meerdink et al.^28^, Ely et al.^29^ and Wang et al.^17^, involves random partitioning of the full data set into a calibration set (typically 80%) and a validation set (20%). Within the calibration set, 70% of the samples are randomly selected to train the model using varying values of NoC, while the remaining 30% of the calibration set are used to assess model performance of each NoC. This procedure is typically repeated 1000 times to ensure robust estimation. The optimal NoC value is chosen by minimizing the predicted residual error sum of squares (PRESS) statistics^30^ collected from the repeated sub-sampling. The second approach, known as repeated double cross-validation, employs two nested cross-validations, and has been utilized in studies such as Dechant et al.^31^, Yan et al.^32^ and Ji et al.^33^.

In this study, we compared these two approaches based on both calibration and validation performance. For the first approach a fixed design of 20 repetitions of 5-fold CV was implemented and ${\mathrm{No}}{{\mathrm{C}}_{{\mathrm{MSE}}\left( {{\mathrm{opt}}} \right)}}$ was selected as the optimal NoC for each repetition, while the most frequently chosen value across repetitions was the final estimated NoC: $No{C_{MSE}}$^26^. For the second approach, 100 random 80/20 training-testing sub-sampling was employed to calculate PRESS statistic^30^ and select the optimal NoC.

### Different prediction scenarios

The assessment of the model generalizability to unseen samples has been approached in different ways. For instance, Burnett et al.^25^ assessed the uncertainty of PLSR models using jackknife (leave-on-out) of the calibration data set, given that a single final validation set was available. In contrast, Filzmoser et al.^26^ proposed repeated cross-validation sets, allowing thorough assessment of both prediction accuracy and performance variability. It is then conceivable that these differences can have impact on the findings and their comparability between different studies.

In our study, we adopted the second approach to systematically evaluated the utility of HSR data for predicting physiological traits under three distinct prediction scenarios, each designed to assess model performance under varying levels of data partitioning. Model performance was assessed using the outer loop of a repeated CV framework, while hyperparameter tuning was conducted within the inner loop, as illustrated in Fig. 1a. The scenarios are described in the following:

Scenario i: Random sample prediction. In this scenario, model generalizability was evaluated using randomly partitioned data sets. This approach enables systematic comparison of model accuracy across different data aggregation strategies and seasons. The outer loop consists of 20 repetitions of random 5-fold CV, allowing the collection of mean performance from five folds across repetitions. Hyperparameter optimization within the inner loop was conducted using either repeated CV, permutation-based, or single CV approaches.

Scenario ii: Unseen genotype prediction. A 5-fold CV scheme was repeated 20 times, ensuring genotype exclusivity between folds in both outer and inner loops to avoid data leakage. This set-up allowed for the assessment of the model’s transferability across genotypes.

Scenario iii: Unseen season prediction. In this scenario, the goal was to predict traits for a new, unseen season using data from other seasons in model training. Here, entire data sets from one or two seasons were used for model calibration, and model performance was evaluated on data from the held-out season. Within the calibration set, the inner loop employed random splits for hyperparameter tuning. Additionally, a more stringent variation of this scenario was implemented, in which the model was trained on data from a single season and was then applied to unseen genotypes in an unseen season. This was achieved by withholding 1/5 of the data of the genotypes from the training season and evaluating their predictions in a separate season in the framework of 20 repetitions of 5-fold CV. Here, too, genotype exclusion was strictly enforced during inner loop splitting to avoid data leakage.

Model performance was quantified by using coefficient of determination (R^2^) for scenarios i and ii and squared Pearson correlation for scenario iii. We used squared Pearson correlation to assess the trend of predicted values rather than their agreement on measured values in the most challenging scenario iii.

## Supplementary Information

Below is the link to the electronic supplementary material.

Supplementary Material 1

Supplementary Material 2

Supplementary Material 3

## Data Availability

All code and raw data to ensure reproducibility of the results can be accessed at: [https://github.com/Rudan-X/HyperspectralML](https:/github.com/Rudan-X/HyperspectralML).

## Abbreviations

HSR: Hyperspectral reflectance
PLSR: Partial least squares regression
NoC: Number of component
SVR: Support vector regression
*A-Ci* curve: Photosynthetic rate versus internal CO_2_ concentration curve
PEPC: Phosphoenolpyruvate carboxylase
V_pmax_: Maximum rate of carboxylation by PEPC
V_max_: Asymptote of the *A-Ci* curve
PSII: Photosystem II
Fv/Fm: Quantum efficiency of PSII
NPQ: Non-photochemical quenching.
ΦPSII: PSII operating efficiency
%C: Relative carbon content
%N: Relative nitrogen content
δ^13^C: Stable carbon isotope ratio
δ^15^N: Stable nitrogen isotope ratio
iWUE: Intrinsic water use efficiency
gsw: Stomatal conductance
R^2^: Coefficient of determination
CV: Cross-validation
CVs: Coefficients of variation
SD: Standard deviation

## References

[1] Kole, C. et al. Application of genomics-assisted breeding for generation of climate resilient crops: progress and prospects. *Front. Plant Sci.* **6**, 563 (2015). 26322050 10.3389/fpls.2015.00563 PMC4531421

[2] Langridge, P., Braun, H., Hulke, B., Ober, E. & Prasanna, B. Breeding crops for climate resilience. *Theor. Appl. Genet.* **134**, 1607–1611 (2021). 34046700 10.1007/s00122-021-03854-7

[3] Alseekh, S., Kostova, D., Bulut, M. & Fernie, A. R. Genome-wide association studies: assessing trait characteristics in model and crop plants. *Cell. Mol. Life Sci.* **78**, 5743–5754 (2021). 34196733 10.1007/s00018-021-03868-w PMC8316211

[4] Grzybowski, M., Wijewardane, N. K., Atefi, A., Ge, Y. & Schnable, J. C. Hyperspectral reflectance-based phenotyping for quantitative genetics in crops: progress and challenges. *Plant Commun.* **2**, 145 (2021). 10.1016/j.xplc.2021.100209 PMC8299078 34327323

[5] Magney, T. S. Hyperspectral reflectance integrates key traits for predicting leaf metabolism. *New Phytol.* **246**, 383 (2024). 39673249 10.1111/nph.20345 PMC11923394

[6] Ye, X., Abe, S. & Zhang, S. Estimation and mapping of nitrogen content in Apple trees at leaf and canopy levels using hyperspectral imaging. *Precision Agric.* **21**, 198–225 (2020).

[7] Qian, T., Elings, A., Dieleman, J. A., Gort, G. & Marcelis, L. F. M. Estimation of photosynthesis parameters for a modified Farquhar–von Caemmerer–Berry model using simultaneous estimation method and nonlinear mixed effects model. *Environ. Exp. Bot.* **82**, 66–73 (2012).

[8] Kumar, L., Schmidt, K., Dury, S. & Skidmore, A. Imaging spectrometry and vegetation science. *Imaging Spectrometry: Basic Principles Prospect. Appl.* **2001**, 111–155 (2001).

[9] Serbin, S. P., Dillaway, D. N., Kruger, E. L. & Townsend, P. A. Leaf optical properties reflect variation in photosynthetic metabolism and its sensitivity to temperature. *J. Exp. Bot.* **63**, 489–502 (2012). 21984647 10.1093/jxb/err294 PMC3245480

[10] Yendrek, C. R. et al. High-throughput phenotyping of maize leaf physiological and biochemical traits using hyperspectral reflectance. *Plant Physiol.* **173**, 614–626 (2017). 28049858 10.1104/pp.16.01447 PMC5210743

[11] Heckmann, D., Schlüter, U. & Weber, A. P. Machine learning techniques for predicting crop photosynthetic capacity from leaf reflectance spectra. *Mol. Plant*. **10**, 878–890 (2017). 28461269 10.1016/j.molp.2017.04.009

[12] Silva-Perez, V. et al. Hyperspectral reflectance as a tool to measure biochemical and physiological traits in wheat. *J. Exp. Bot.* **69**, 483–496 (2018). 29309611 10.1093/jxb/erx421 PMC5853784

[13] Cotrozzi, L., Peron, R., Tuinstra, M. R., Mickelbart, M. V. & Couture, J. J. Spectral phenotyping of physiological and anatomical leaf traits related with maize water status. *Plant Physiol.* **184**, 1363–1377 (2020). 32907885 10.1104/pp.20.00577 PMC7608158

[14] Feng, X. et al. Hyperspectral imaging combined with machine learning as a tool to obtain high-throughput plant salt-stress phenotyping. *Plant J.* **101**, 1448–1461 (2020). 31680357 10.1111/tpj.14597

[15] Rehman, T. U., Ma, D., Wang, L., Zhang, L. & Jin, J. Predictive spectral analysis using an end-to-end deep model from hyperspectral images for high-throughput plant phenotyping. *Comput. Electron. Agric.* **177**, 105713 (2020).

[16] Furbank, R. T. et al. Wheat physiology predictor: predicting physiological traits in wheat from hyperspectral reflectance measurements using deep learning. *Plant. Methods*. **17**, 1–15 (2021). 34666801 10.1186/s13007-021-00806-6 PMC8527791

[17] Wang, S. et al. Unique contributions of chlorophyll and nitrogen to predict crop photosynthetic capacity from leaf spectroscopy. *J. Exp. Bot.* **72**, 341–354 (2021). 32937655 10.1093/jxb/eraa432

[18] Yu, S. et al. Hyperspectral technique combined with deep learning algorithm for prediction of phenotyping traits in lettuce. *Front. Plant Sci.* **13**, 927832 (2022). 35845657 10.3389/fpls.2022.927832 PMC9279906

[19] Kaur, S., Kakani, V. G., Carver, B., Jarquin, D. & Singh, A. Hyperspectral imaging combined with machine learning for high-throughput phenotyping in winter wheat. *Plant. Phenome J.* **7**, e20111 (2024).

[20] Kyaw, T. Y. et al. Using hyperspectral leaf reflectance to estimate photosynthetic capacity and nitrogen content across Eastern cottonwood and hybrid Poplar taxa. *Plos One*. **17**, e0264780 (2022). 35271605 10.1371/journal.pone.0264780 PMC8912144

[21] Meacham-Hensold, K. et al. High-throughput field phenotyping using hyperspectral reflectance and partial least squares regression (PLSR) reveals genetic modifications to photosynthetic capacity. *Remote Sens. Environ.* **231**, 111176 (2019). 31534277 10.1016/j.rse.2019.04.029 PMC6737918

[22] Ge, Y. et al. High-throughput analysis of leaf physiological and chemical traits with VIS–NIR–SWIR spectroscopy: a case study with a maize diversity panel. *Plant. Methods*. **15**, 1–12 (2019). 31391863 10.1186/s13007-019-0450-8 PMC6595573

[23] Yamashita, H., Sonobe, R., Hirono, Y., Morita, A. & Ikka, T. Dissection of hyperspectral reflectance to estimate nitrogen and chlorophyll contents in tea leaves based on machine learning algorithms. *Sci. Rep.* **10**, 17360 (2020). 33060629 10.1038/s41598-020-73745-2 PMC7566634

[24] Fu, P., Meacham-Hensold, K., Guan, K. & Bernacchi, C. J. Hyperspectral leaf reflectance as proxy for photosynthetic capacities: an ensemble approach based on multiple machine learning algorithms. *Front. Plant Sci.* **10**, 730 (2019). 31214235 10.3389/fpls.2019.00730 PMC6556518

[25] Burnett, A. C. et al. A best-practice guide to predicting plant traits from leaf-level hyperspectral data using partial least squares regression. *J. Exp. Bot.* **72**, 6175–6189 (2021). 34131723 10.1093/jxb/erab295

[26] Filzmoser, P., Liebmann, B. & Varmuza, K. Repeated double cross validation. *J. Chemometrics: J. Chemometrics Soc.* **23**, 160–171 (2009).

[27] Serbin, S. P., Singh, A., McNeil, B. E., Kingdon, C. C. & Townsend, P. A. Spectroscopic determination of leaf morphological and biochemical traits for Northern temperate and boreal tree species. *Ecol. Appl.* **24**, 1651–1669 (2014). 10.1890/13-2110.1 29210229

[28] Meerdink, S. K. et al. Linking seasonal foliar traits to VSWIR-TIR spectroscopy across California ecosystems. *Remote Sens. Environ.* **186**, 322–338 (2016).

[29] Ely, K. S., Burnett, A. C., Lieberman-Cribbin, W., Serbin, S. P. & Rogers, A. Spectroscopy can predict key leaf traits associated with source–sink balance and carbon–nitrogen status. *J. Exp. Bot.* **70**, 1789–1799 (2019). 30799496 10.1093/jxb/erz061

[30] Chen, S., Hong, X., Harris, C. J. & Sharkey, P. M. Sparse modeling using orthogonal forward regression with PRESS statistic and regularization. *IEEE Trans. Syst. Man. Cybern. Part. B (Cybern.)* **34**, 898–911 (2004). 10.1109/tsmcb.2003.817107 15376838

[31] Dechant, B., Cuntz, M., Vohland, M., Schulz, E. & Doktor, D. Estimation of photosynthesis traits from leaf reflectance spectra: correlation to nitrogen content as the dominant mechanism. *Remote Sens. Environ.* **196**, 279–292 (2017).

[32] Yan, Z. et al. Spectroscopy outperforms leaf trait relationships for predicting photosynthetic capacity across different forest types. *New Phytol.* **232**, 134–147 (2021). 34165791 10.1111/nph.17579

[33] Ji, F. et al. Unveiling the transferability of PLSR models for leaf trait estimation: lessons from a comprehensive analysis with a novel global dataset. *New Phytol.* **243**, 111–131 (2024). 38708434 10.1111/nph.19807

[34] Tibshirani, R. & Friedman, J. *The Elements of Statistical Learning: Data Mining, Inference, and Prediction* (Springer, 2001).

[35] Ferguson, J. N. et al. A deficient CP24 allele defines variation for dynamic nonphotochemical quenching and photosystem II efficiency in maize. *Plant. Cell.* **37**, koaf063 (2025). 40132112 10.1093/plcell/koaf063 PMC12018801

[36] Dell’Acqua, M. et al. Genetic properties of the MAGIC maize population: a new platform for high definition QTL mapping in Zea Mays. *Genome Biol.* **16**, 1–23 (2015). 26357913 10.1186/s13059-015-0716-z PMC4566846

[37] Ferguson, J. N., Jithesh, T., Lawson, T. & Kromdijk, J. Excised leaves show limited and species-specific effects on photosynthetic parameters across crop functional types. *J. Exp. Bot.* **74**, 6662–6676 (2023). 37565685 10.1093/jxb/erad319 PMC10662226

[38] Ferguson, J. N. et al. Reducing stomatal density by expression of a synthetic epidermal patterning factor increases leaf intrinsic water use efficiency and reduces plant water use in a C4 crop. *J. Exp. Botany* **2024**, erae289 (2024). 10.1093/jxb/erae289 PMC11565208 39021331

[39] Von Caemmerer, S. *Biochemical Models of Leaf Photosynthesis* (Csiro publishing, 2000).

[40] Mevik, B. H. & Wehrens, R. The Pls package: principal component and partial least squares regression in R. *J. Stat. Softw.* **18**, 1–24 (2023).

[41] Kuhn & Max. Building predictive models in R using the caret package. *J. Stat. Softw.* **28**, 1–26 (2008).27774042

[42] Geladi, P. & Kowalski, B. R. Partial least-squares regression: a tutorial. *Anal. Chim. Acta*. **185**, 1–17 (1986).

[43] Wold, S., Sjöström, M. & Eriksson, L. PLS-regression: a basic tool of chemometrics. *Chemometr. Intell. Lab. Syst.* **58**, 109–130 (2001).

