# Supplementary material for: Generalizability and transferability of machine learning models using hyperspectral reflectance data for maize traits
Source: Sci Rep. 2026 Jan 21;16:5865. doi: 10.1038/s41598-026-36819-1 (PMC12894961; doi:10.1038/s41598-026-36819-1)
Supplement: Supplementary file 3 — Supplementary Material 3 [file 41598_2026_36819_MOESM3_ESM.docx]

**Generalizability and transferability of machine learning models using hyperspectral reflectance data for maize traits**

Rudan Xu^1,2,†^, John Ferguson^3,†^, Matthieu Breil-Aubert^4^, Johannes Kromdijk^4,*^, and Zoran Nikoloski^1,2, *^

^1^Bioinformatics Department, Institute of Biochemistry and Biology, University of Potsdam, Potsdam, Germany

^2^Systems Biology and Mathematical Modelling Group, Max Planck Institute of Molecular

Plant Physiology, Potsdam, Germany

^3^School of Life Sciences, University of Essex, Colchester, UK

^4^Department of Plant Sciences, University of Cambridge, Cambridge, UK

^†^These authors contributed equally.

^*^Corresponding authors.

**Email address:**

xu2@uni-potsdam.de

jfergu@essex.ac.uk

mb2651@cam.ac.uk

jk417@cam.ac.uk

nikoloski@mpimp-golm.mpg.de

**Supplementary Figures**


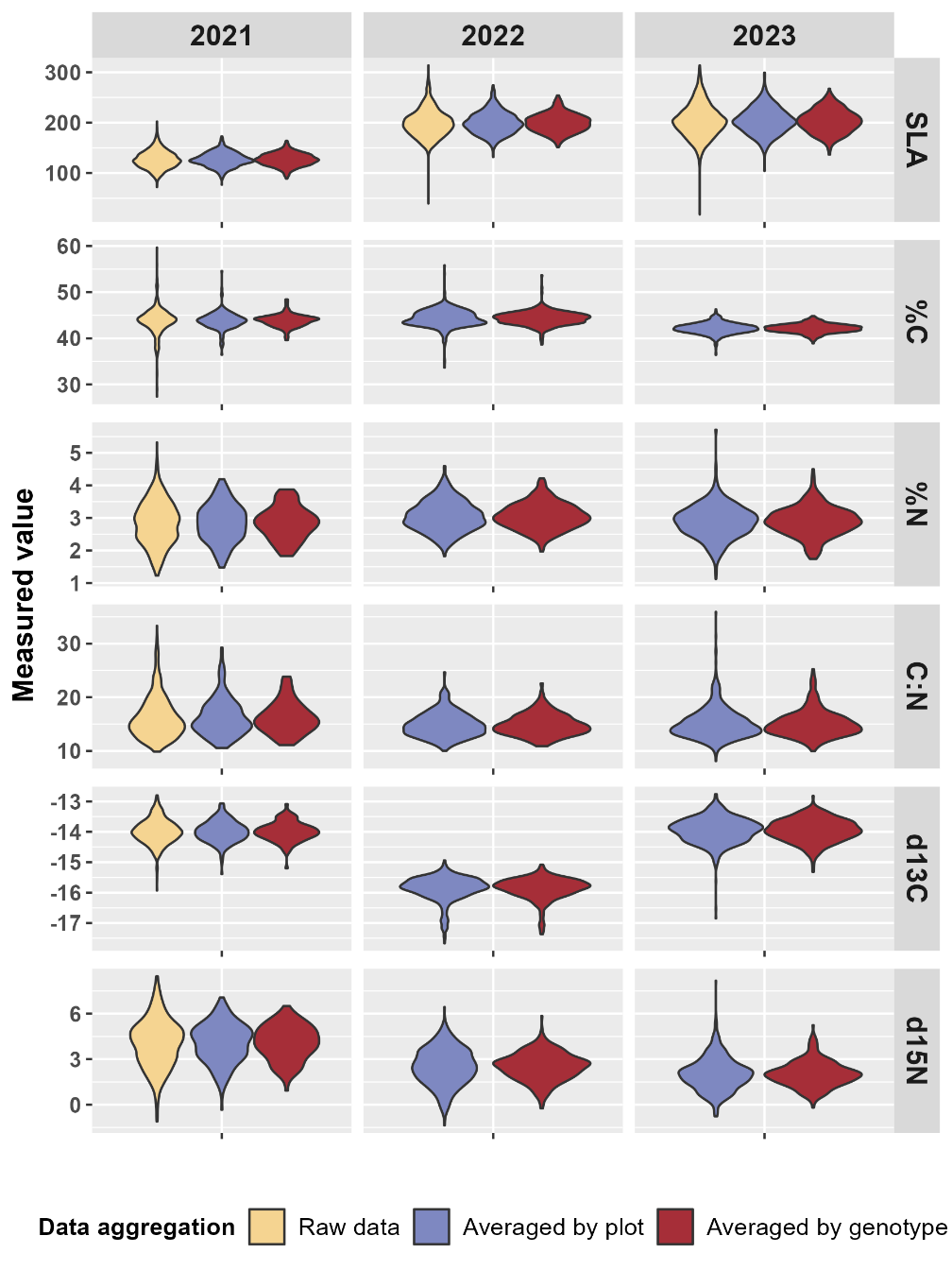


**Figure S1. Distribution patterns of structural and biochemical traits across three consecutive growing seasons (2021–2023).** Violin plots illustrate the variability and distribution of six key structural and biochemical traits: specific leaf area (SLA), percentage nitrogen (%N), percentage carbon (%C), carbon-to-nitrogen ratio (C/N), nitrogen isotope ratio (δ15N), and carbon isotope ratio (δ13C). Data are presented at three aggregation levels: raw data (yellow), averaged by plot (blue), and averaged by genotype (red).

**
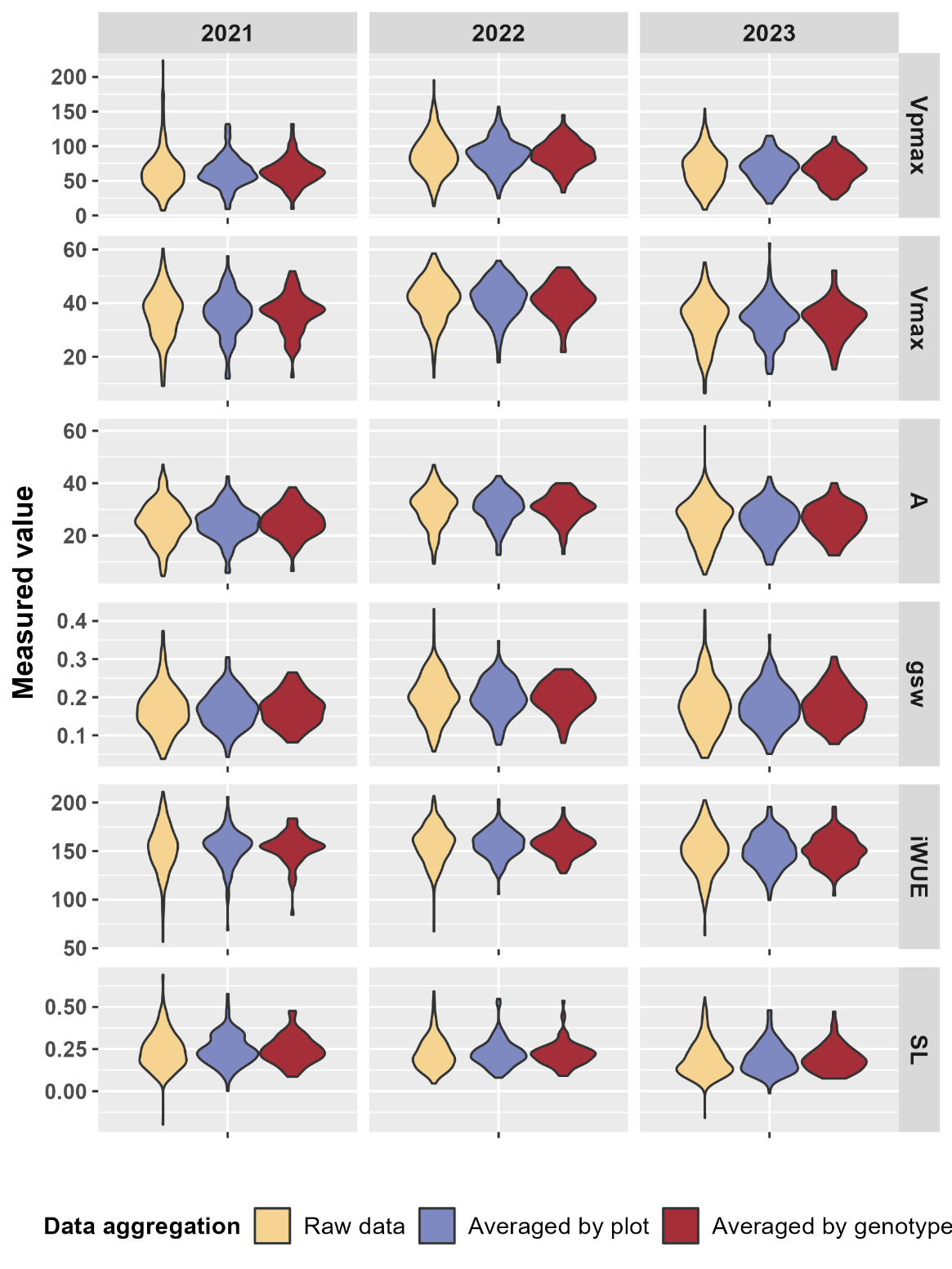
**

**Figure S2. Distribution patterns of photosynthetic traits over three consecutive growing seasons (2021–2023).** Violin plots illustrate variability and distributions of seven photosynthetic traits derived from gas exchange measurements: maximum carboxylation rate (V_max_), maximum electron transport rate (V_pmax_), intrinsic water-use efficiency (iWUE), stomatal conductance (Gsw), photosynthesis rate at saturating light (A), and stomatal limitation (SL) at saturating light and ambient CO_2_.

**
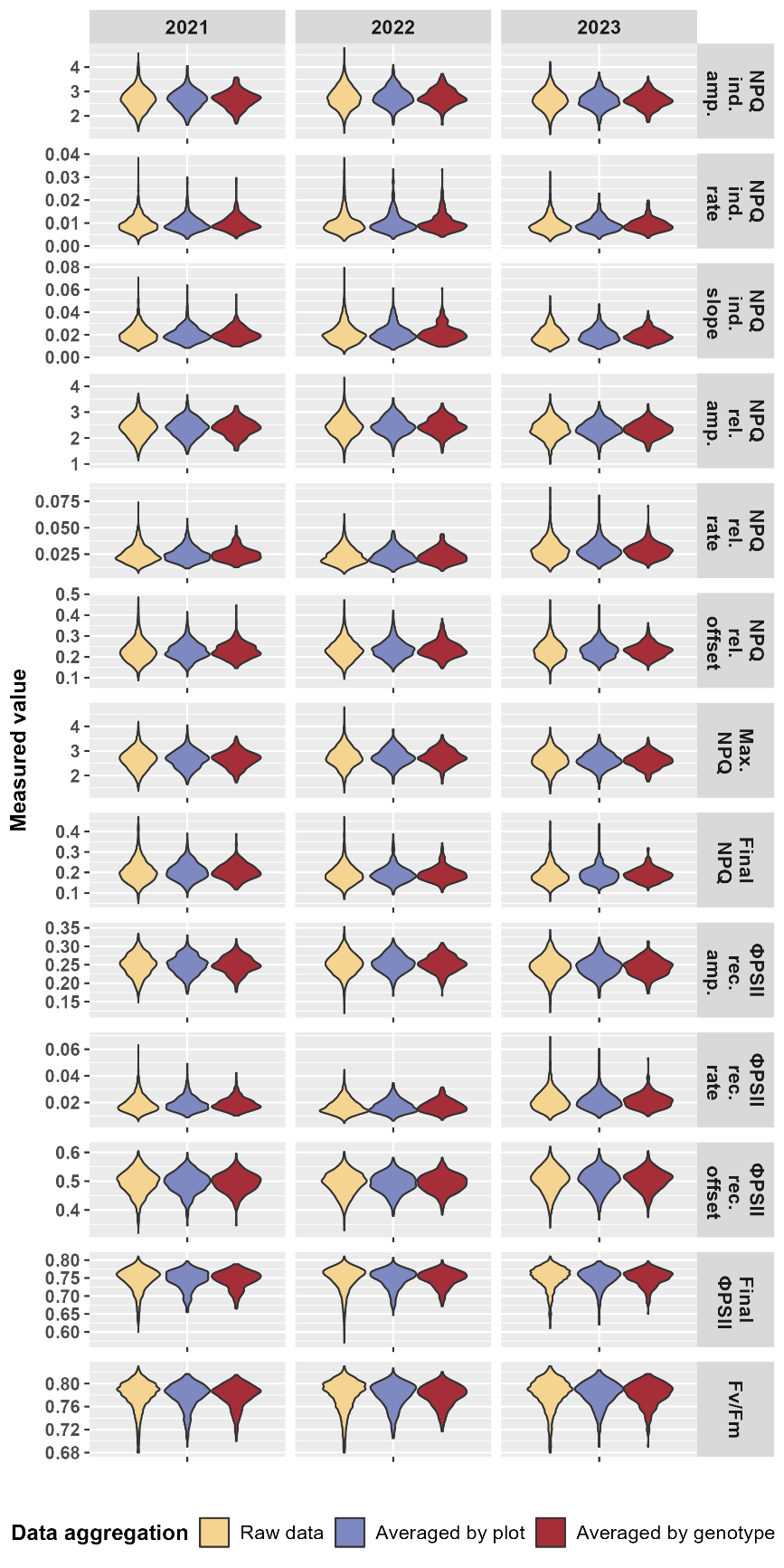
**

**Figure S3. Distribution patterns of chlorophyll fluorescence traits over three consecutive growing seasons (2021–2023).** Violin plots illustrate variability and distributions of thirteen chlorophyll fluorescence traits associated with photosystem II performance, including quantum efficiency (Fv/Fm), photochemical quenching efficiency (ΦPSII) and non-photochemical quenching (NPQ).

*
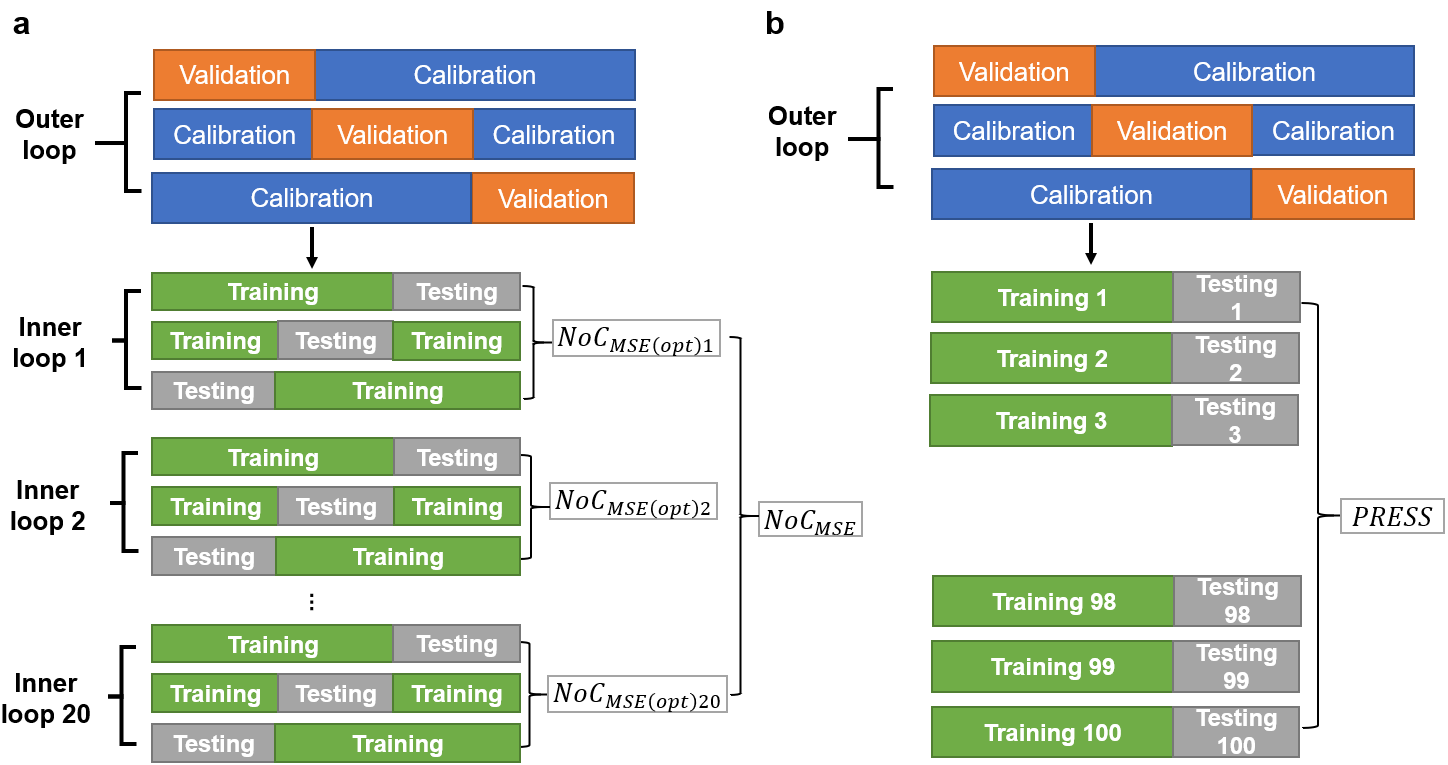
*

**Figure S4. Illustration of two different calibration metrics and the corresponding calibration procedure.** The outer-loop partitions the data into a calibration set (blue) and a validation set (orange). (a) For the MSE-based approach, the calibration set is further split into K-fold CV in the inner loop. For each testing fold (gray), NoC values ranging from 1 to 40 are used to train the model (green) and tested using MSE metric. The optimal NoC for that repetition, $NoC_{MSE(opt)}$, is defined as the NoC value yielding the smallest MSE across the K folds. This inner loop procedure is repeated 20 times, resulting in 20 $NoC_{MSE(opt)}$ values. The final consensus NoC, ${NoC}_{MSE}$, is the most frequently selected value across the 20 repetitions. When only a single K-fold CV is used, (instead of 20 repeated K-fold), the corresponding $NoC_{MSE(opt)}$ is used as the NoC estimate. (b) For the PRESS-based approach, each calibration set is sub-sampled 100 times: in each repetition, 80% of the data is used for training and 20% for testing. For every NoC, the PRESS value is computed on the testing subset. Repeating this procedure 100 times yields the PRESS statistics used for NoC selection.

**
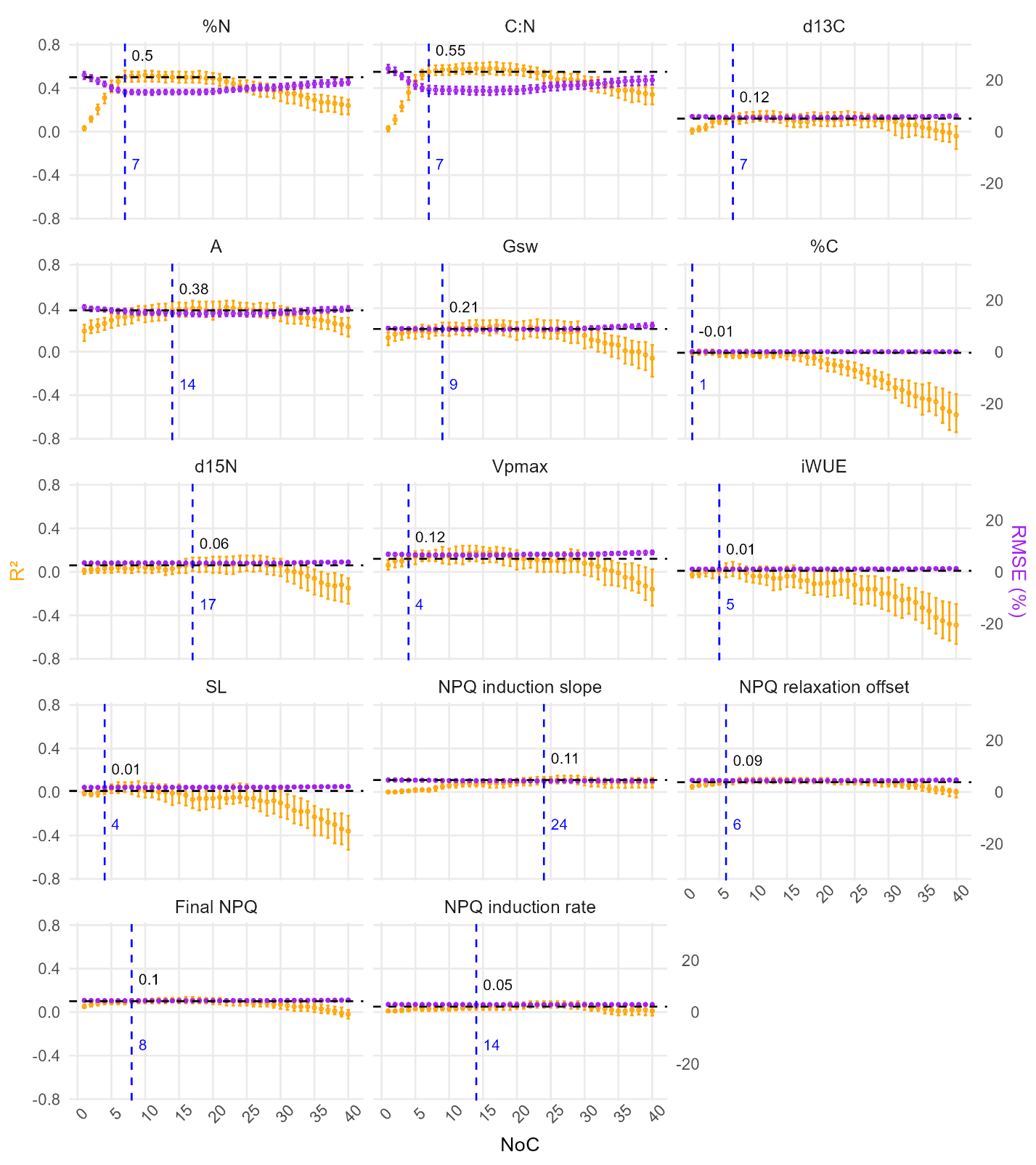
**

**Figure S5. Sensitivity of PLSR model calibration to the number of component (NoC) across remaining traits measured in season 2021.** Each panel shows model performances as a function of NoC values. Model performance was evaluated using 20 repetitions of 5-fold cross-validation across varying NoC values. The coefficient of determination (R^2^, orange, left y-axis) and RMSE% (purple, right y-axis) are shown in dots as median values across the 100 test folds, with error bars representing the interquartile range (25th to 75th percentile). Vertical dashed lines denote the optimal number of components, ${NoC}_{MSE}$, while horizontal dashed lines denote the corresponding median R^2^.


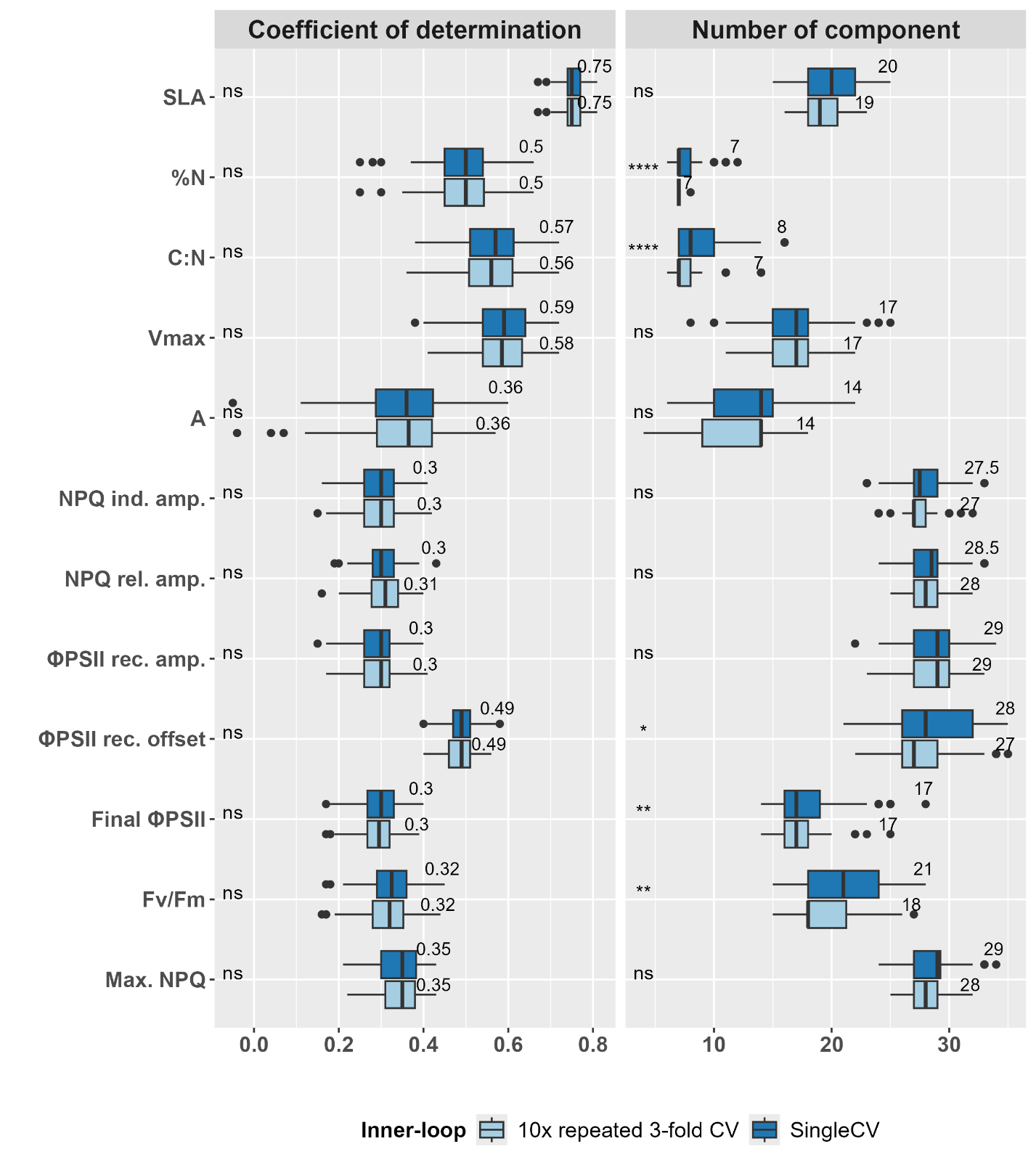


**Figure S6**. **Comparison between inner-loop cross-validation strategy on model performance and optimal NoC estimation.** Comparison between a single CV setup (singleCV) and 10-times repeated 3-fold CV (RepeatedCV) used in the inner loop of PLSR model tuning. Left panel shows the coefficient of determination (R^2^) for traits showing median R^2^ higher than 0.3, while the right panel shows the selected number of components (NoC) under each strategy. Boxes summarize results from 20 outer-loop repetitions of 5-fold CV. Wilcoxon test was applied with statistical significance between the two CV strategies is indicated on the right using asterisks: * (p ≤ 0.05), ** (p ≤ 0.01), *** (p ≤ 0.001), **** (p ≤ 0.0001), ns = not significant.


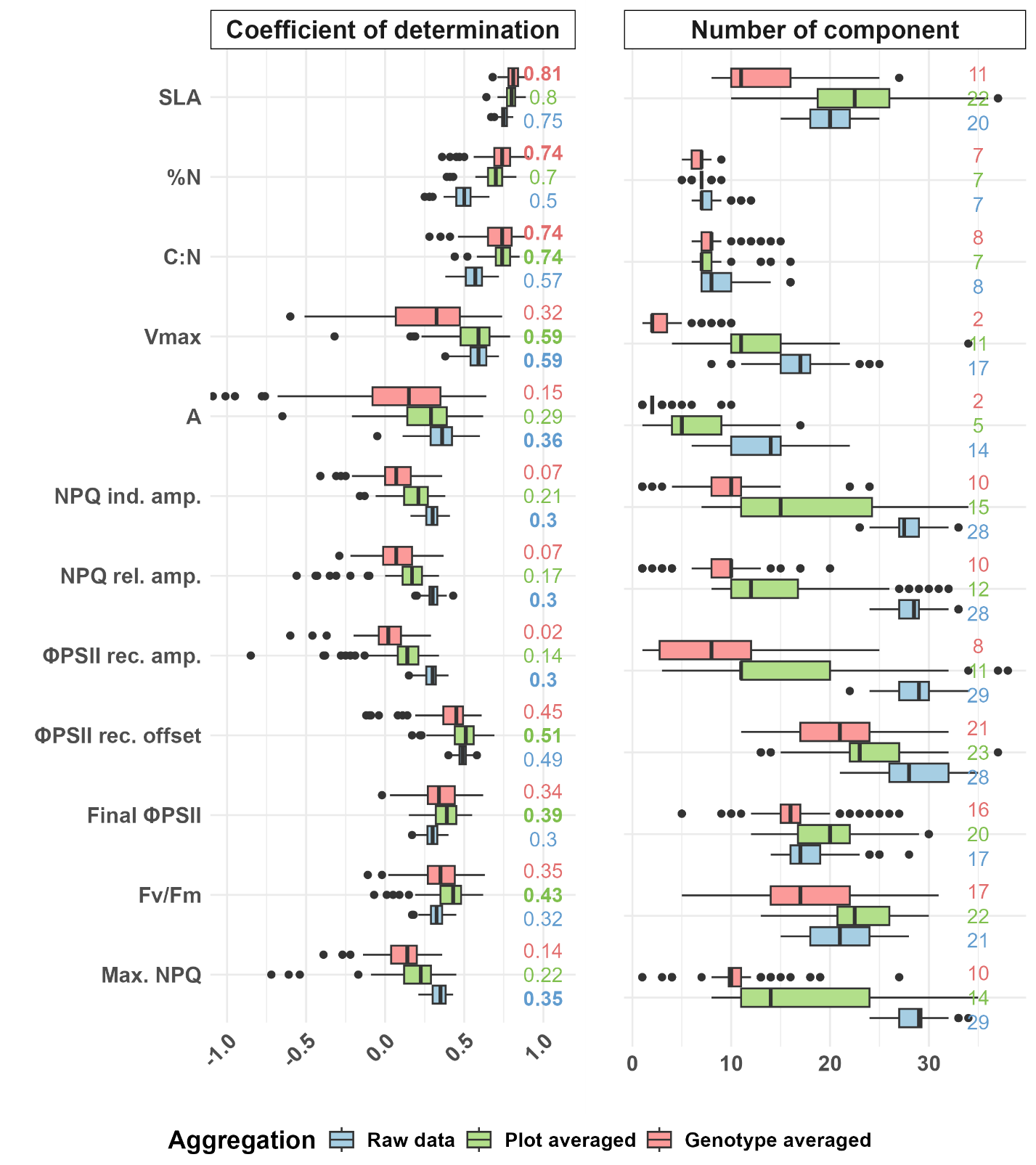


**Figure S7. Effect of aggregation methods on model performance and selected number of components (NoC) for selected traits measured in the 2021 season using PLSR.** The left panel shows boxplots of the coefficient of determination (R^2^) across 20 repetitions of 5-fold CV, with model tuning based on a single inner CV. The right panel displays the corresponding selected NoC in each iteration. Colors indicate the three aggregation strategies: raw data (blue), plot-level averaging (green), and genotype-level averaging (red).

**
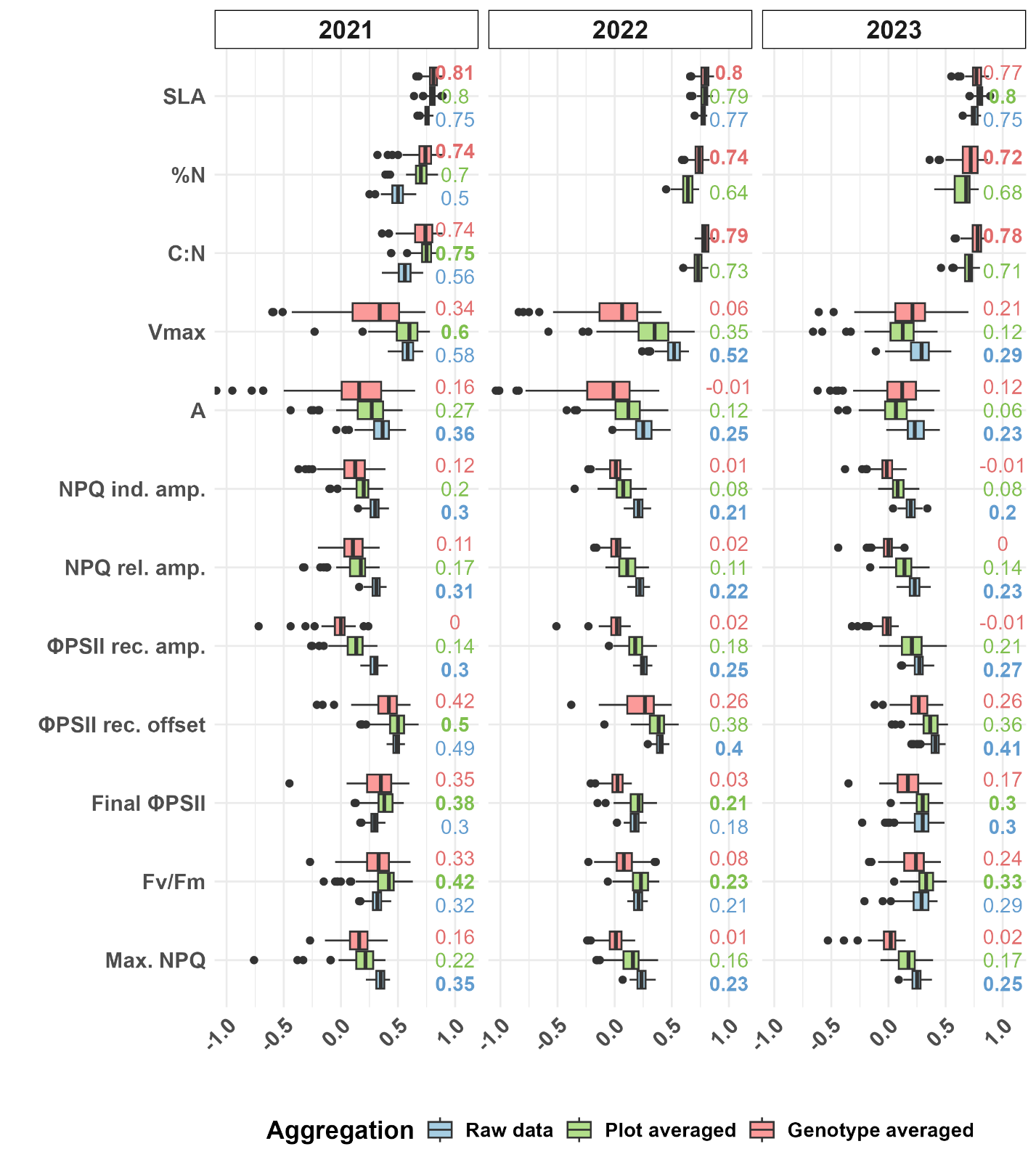
**

**Figure S8. Effect of aggregation methods on model performance and selected number of components (NoC) for traits measured across three seasons using PLSR.** The three panels show boxplots of the coefficient of determination (R^2^) for each season across 20 repetitions of 5-fold CV, with model tuning based on a single inner CV. Colors indicate the three aggregation strategies: raw data (blue), plot-level averaging (green), and genotype-level averaging (red).


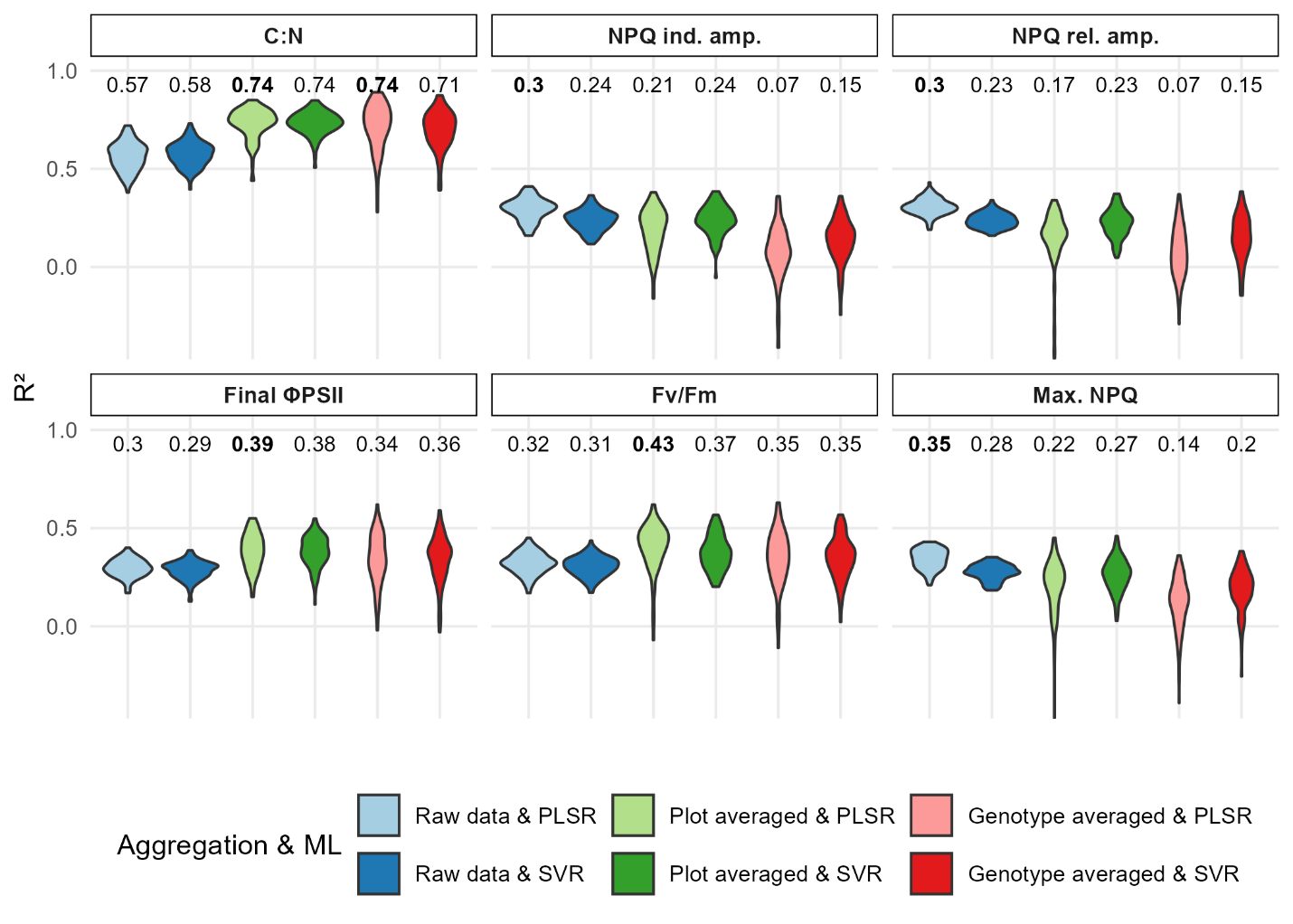


**Figure S9. Comparison of PLSR and SVR model performance under different aggregation strategies for the remaining traits measured in season 2021.** Violin plots depict the distribution of R^2^ scores across 100 validation folds, with median values shown above each plot. Each trait was evaluated using combinations of aggregation methods and machine learning algorithms (color-coded), with model performance assessed via 20 repetitions of 5-fold CV, calibrated with single CV. Bolded numbers indicate the highest median R^2^ achieved among all combinations.

**
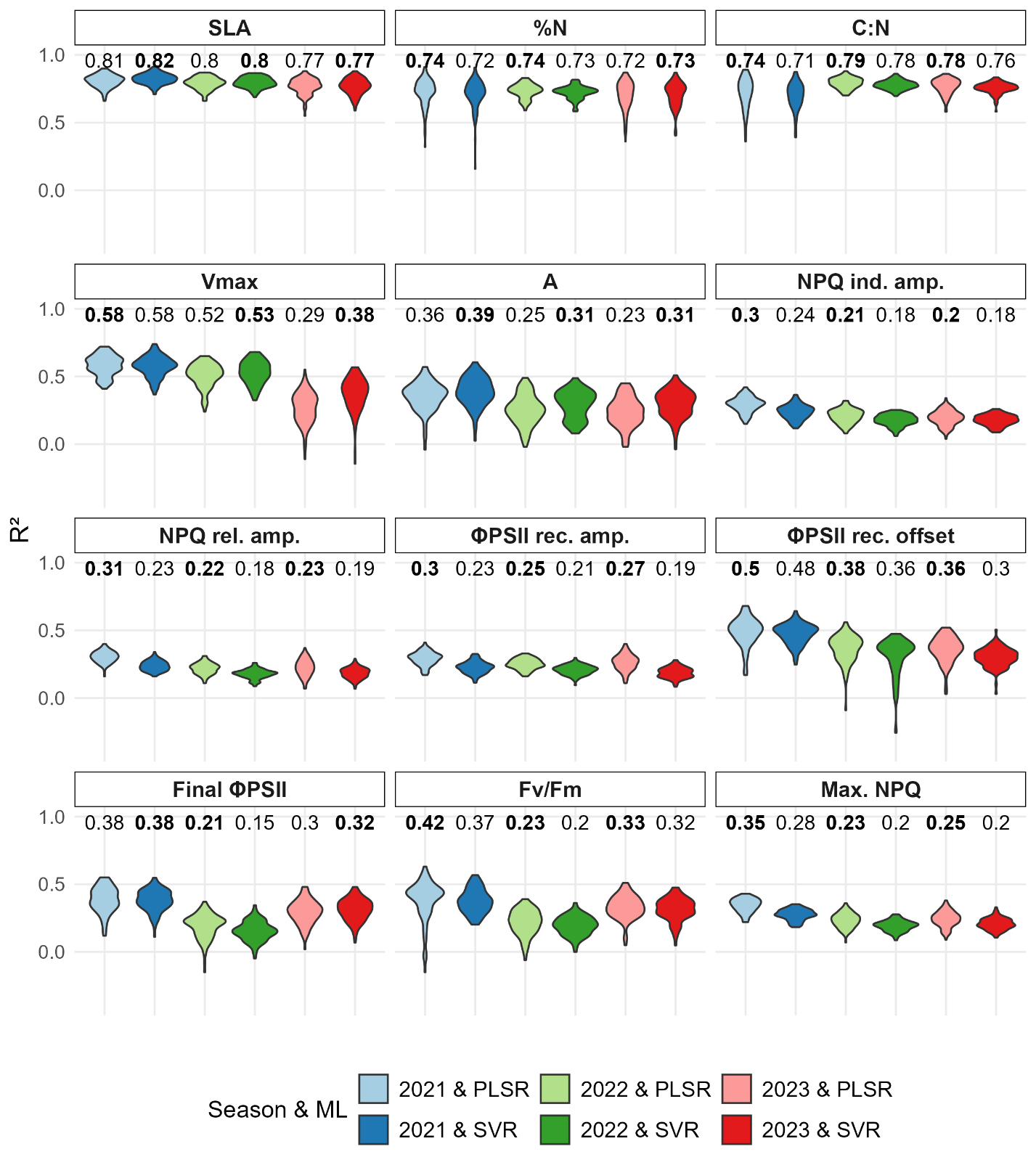
**

**Figure S10. Comparison of PLSR and SVR model performance for 15 traits measured across three seasons, using optimal trait-specific aggregation method.** Violin plots depict the distribution of R^2^ scores across 100 validation folds, with median values shown above each plot. Each trait was predicted using PLSR or SVR (light or dark colored), with model performance assessed via 20 repetitions of 5-fold CV, calibrated with single CV. Bolded numbers indicate the highest median R^2^ achieved among all combinations.

**
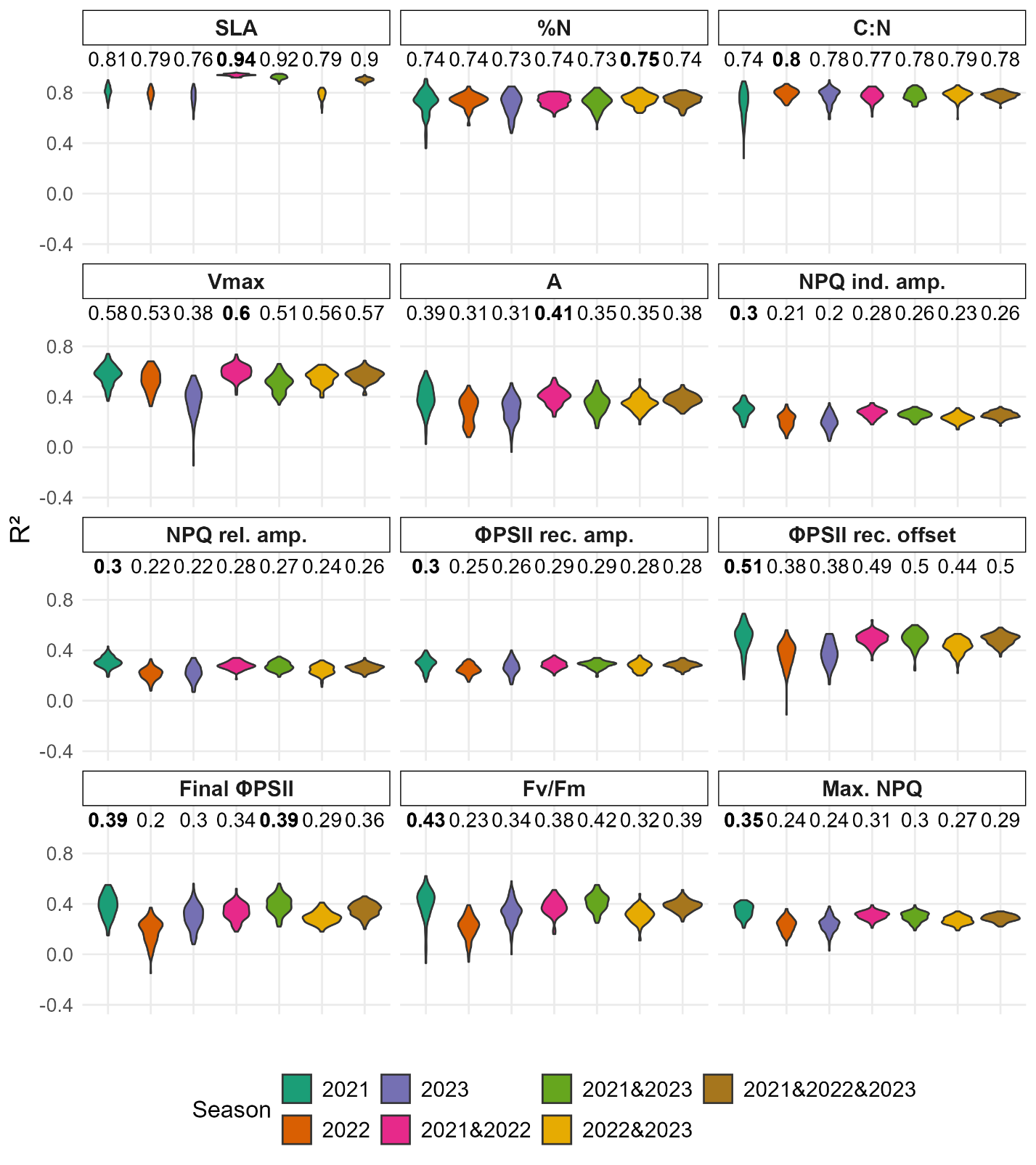
**

**Figure S11. Performance of trait-specific aggregation and ML combination for individual seasons (2021, 2022, and 2023) and all combinations of these seasons.** Violin plots illustrate the R^2^ distribution across 20 repetitions of 5-fold CV, with median values shown above each plot. Bolded numbers indicate the highest median R^2^ achieved among all seasons and their combinations.

**
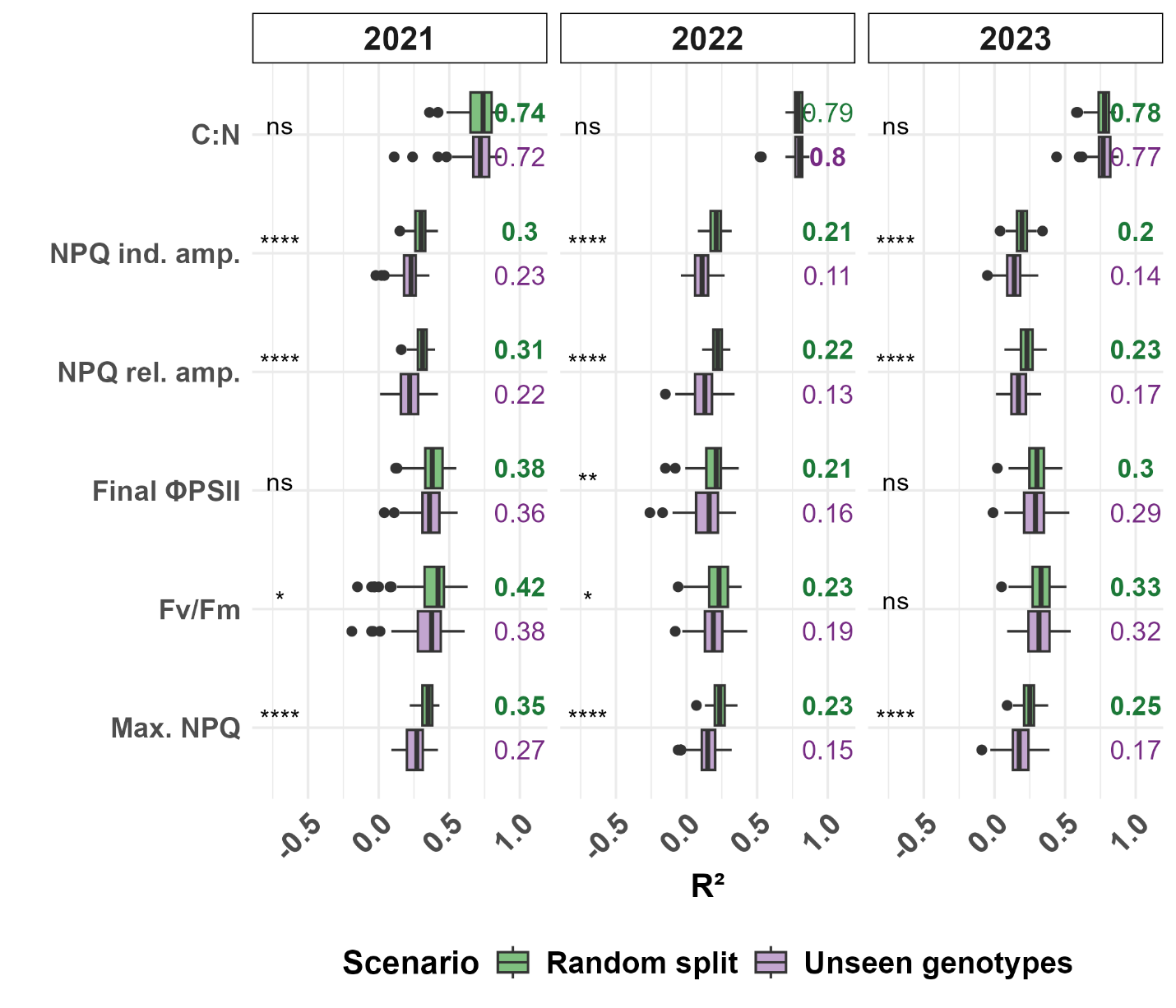
**

**Figure S12. Comparison of prediction performance between random and unseen genotype scenarios using trait-specific combinations of aggregation strategies and machine learning algorithms.** For the remaining six traits measured across three seasons, boxplots show coefficient of determination (R^2^) values across 20 repetitions of 5-fold CV, using the optimal combination of aggregation method and machine learning algorithm for each trait. Two prediction scenarios were evaluated: random data points splitting calibration and validation (green) and validation includes only unseen genotypes (purple). Wilcoxon test was applied with statistical significance between scenarios is indicated by asterisks: * (p ≤ 0.05), ** (p ≤ 0.01), *** (p ≤ 0.001), **** (p ≤ 0.0001), ns = not significant.


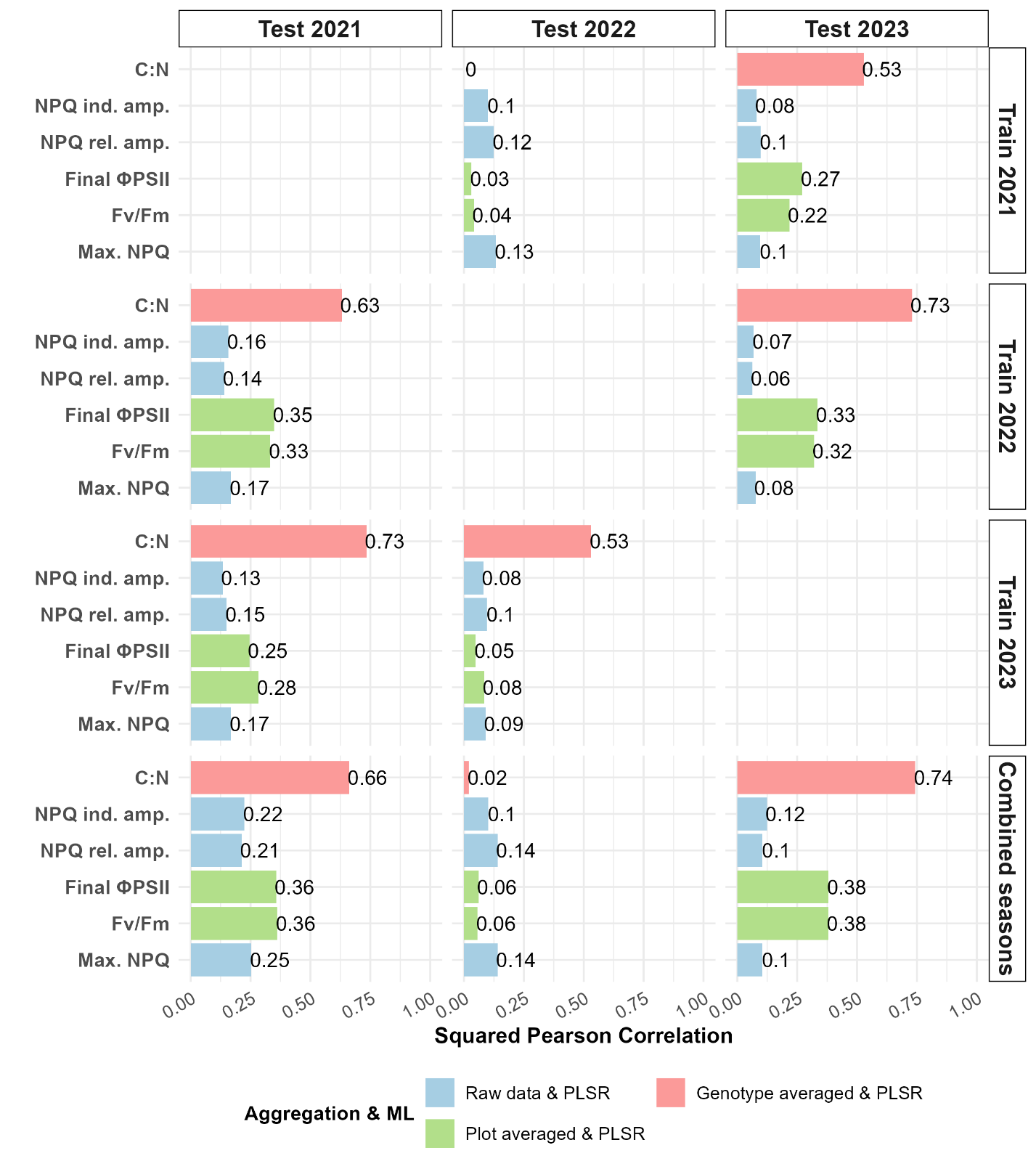


**Figure S13. Prediction performance across unseen seasons using optimal aggregation and ML combinations.** Bar plots display the squared Pearson correlation between predicted and measured trait values for the remaining eleven traits. Rows indicate the season(s) used to calibrate the models (2021, 2022, 2023, or combined seasons), while columns correspond to the test season used for independent validation. For each trait, models were built using the trait-specific optimal combination of aggregation strategy and machine learning algorithm (illustrated in different colors).


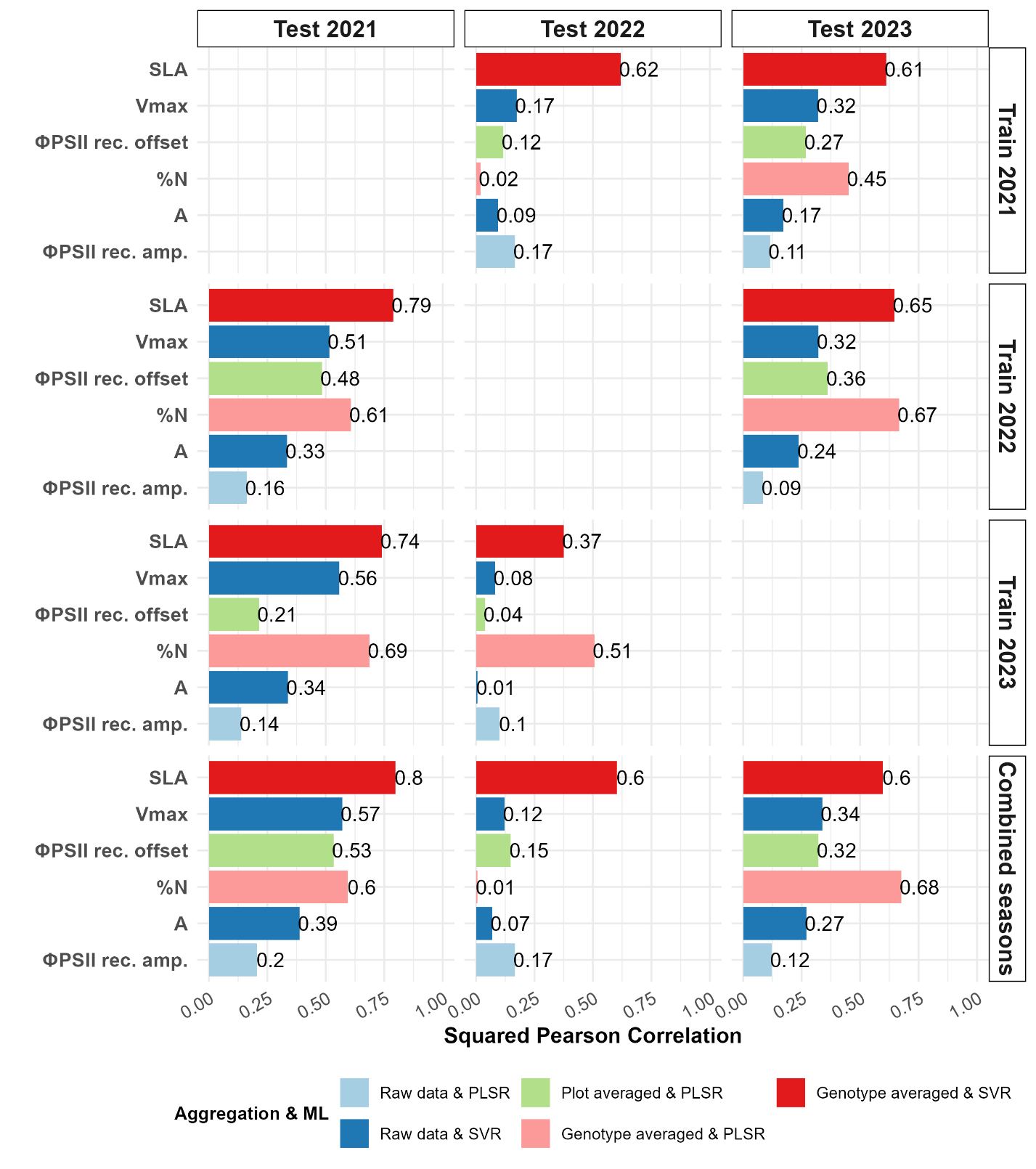


**Figure S14. Prediction performance across unseen seasons using optimal aggregation and ML combinations, applied to scaled data.** Bar plots display the squared Pearson correlation between predicted and measured trait values for the selected six traits. Rows indicate the season(s) used to calibrate the models (2021, 2022, 2023, or combined seasons), while columns correspond to the test season used for independent validation. For each trait, models were built using the trait-specific optimal combination of aggregation strategy and machine learning algorithm (illustrated in different colors).

***
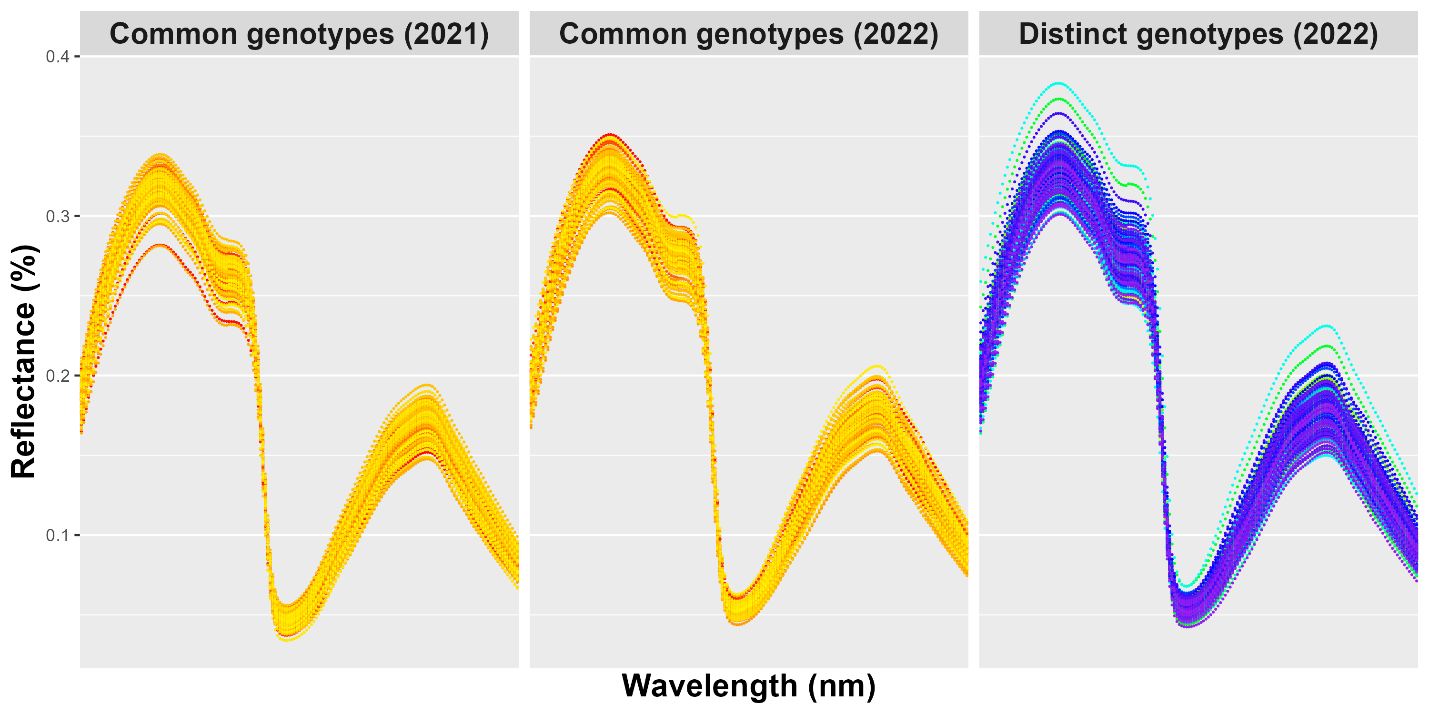
***

**Figure S15. Genotype-averaged HSR profiles corresponding to %N measurements. Spectral profiles show leaf reflectance (%) across the near-infrared spectrum (1500-2400 nm).** Panels differentiate 99 genotypes measured in both seasons (left and middle panels, yellow-red gradient) from 219 genotypes measured only in 2022 (right panel, blue-purple gradient). Each line corresponds to an individual leaf spectrum, illustrating the spectral variability associated with genetic and seasonal differences.

***
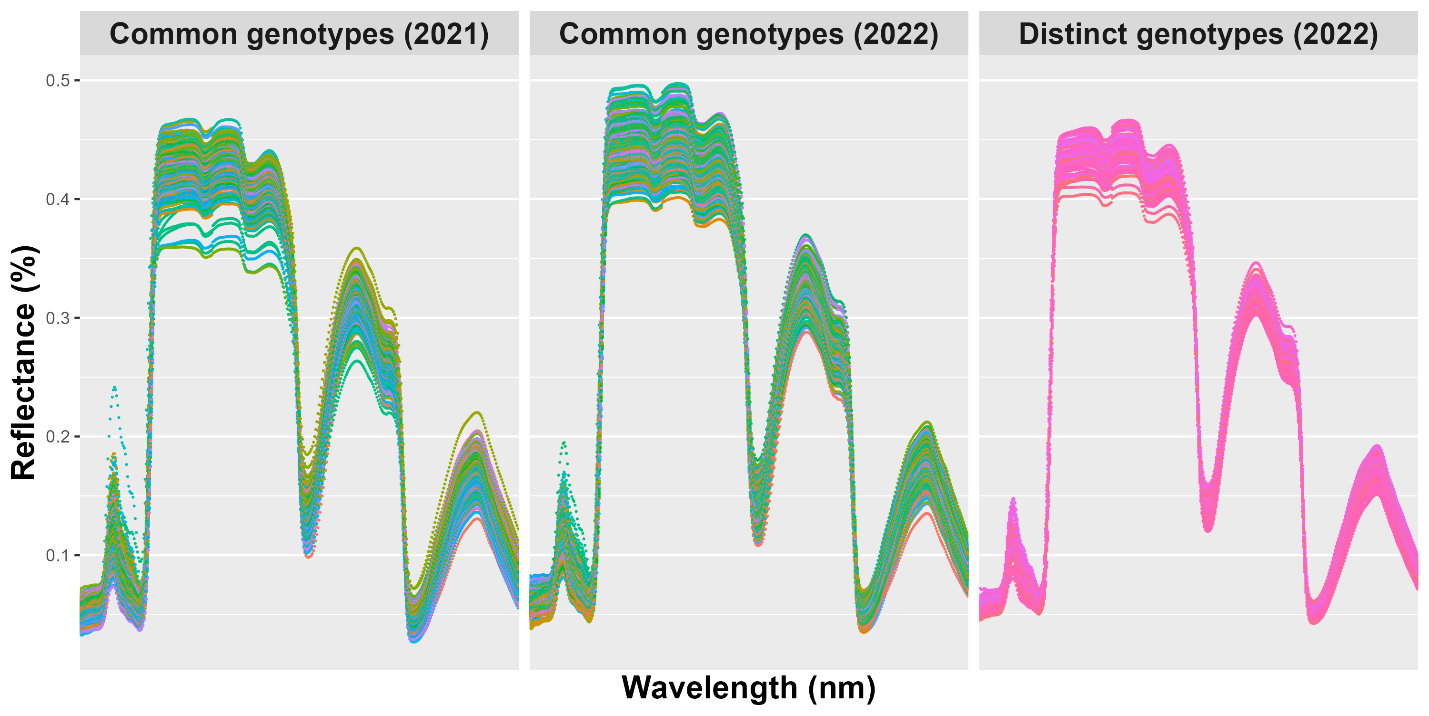
***

**Figure S16. Raw HSR profiles corresponding to gas exchange measurements. Spectral profiles show leaf reflectance (%) in the range between 400 and 2400 nm.** Panels differentiate 69 genotypes measured in both seasons (left and middle panels) from 88 genotypes measured in 2022 (right panel). Each line corresponds to an individual leaf spectrum, illustrating the spectral variability associated with genetic and seasonal differences.


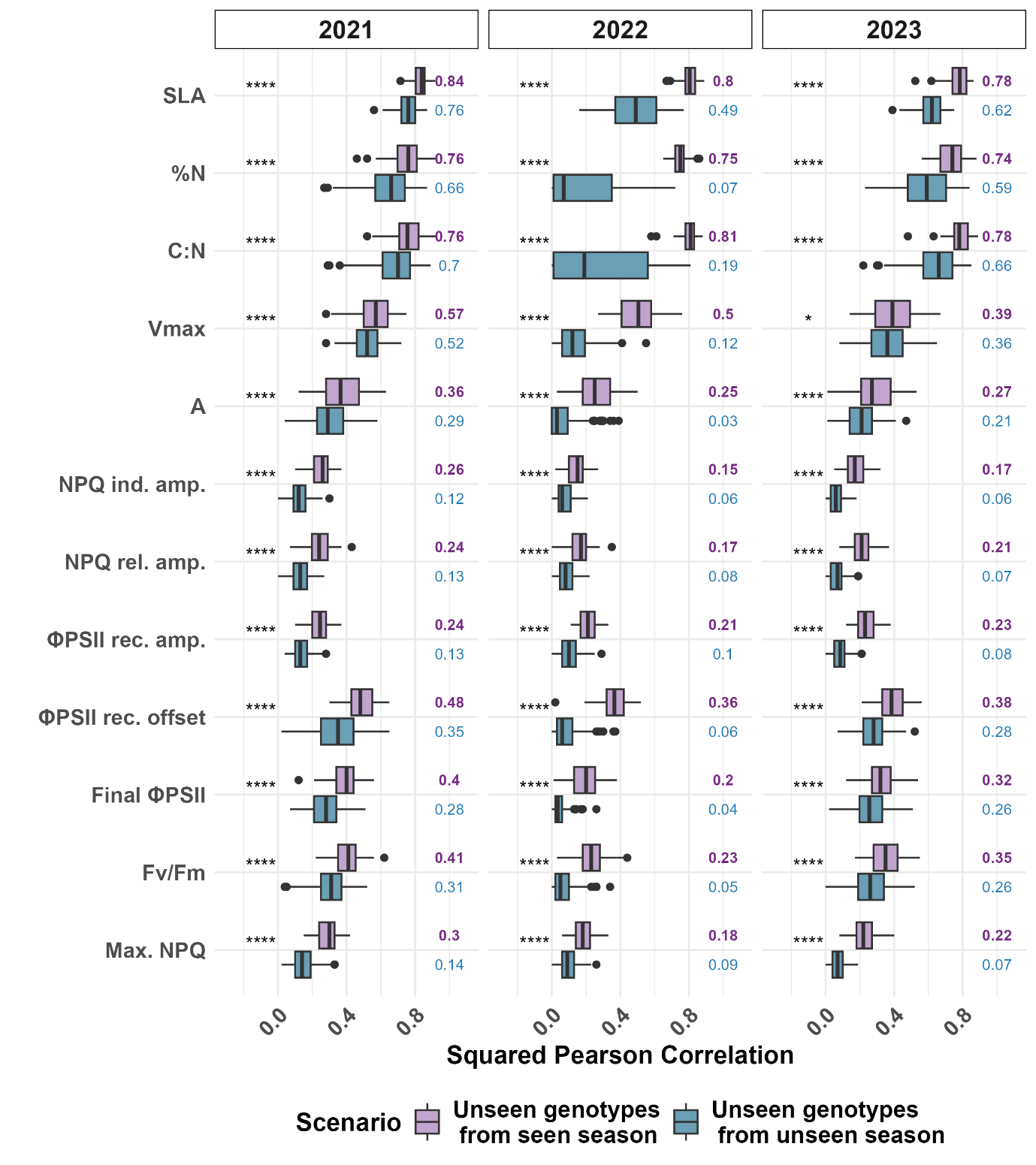


**Figure S17. Comparison of prediction performance between unseen genotypes within the same season and unseen genotypes from unseen seasons using trait-specific aggregation and machine learning strategies.** For twelve traits measured across three seasons, boxplots show square Pearson correlation values across 20 repetitions of 5-fold cross-validation, using the optimal combination of aggregation method and machine learning algorithm for each trait. Two prediction scenarios were evaluated: prediction of unseen genotypes within the same season (blue), and prediction of unseen genotypes from seasons not included in model calibration (purple). Wilcoxon test was applied with statistical significance between scenarios denoted as: * (p ≤ 0.05), *** (p ≤ 0.001), **** (p ≤ 0.0001).

**Supplementary Tables**

**Table S1. Mean Pearson correlation of HSR across different wavelengths between three seasons.**

| **Wavelength range** | **2021&2022** | **2021&2023** | **2022&2023** |
| --- | --- | --- | --- |
| 350-700 nm | 0.273 | 0.367 | 0.296 |
| 700–1300 nm | 0.570 | 0.528 | 0.497 |
| 1300-2500 nm | 0.508 | 0.443 | 0.495 |

**Table S2. Number of raw data samples collected over three consecutive seasons.** The values in parentheses represent the number of samples aggregated by averaging per plot and per genotype.

| **Trait** | **2021** | **2022** | **2023** |
| --- | --- | --- | --- |
| SLA | 1845 (623,316) | 1895 (633,318) | 1669 (556,310) |
| C, N and isotopes | 600 (201,102) | 1069 (624,319) | 936 (618,320) |
| Chlorophyll fluorescence | 1810 (626,316) | 1678 (557,312) | 3312 (586,318) |
| Gas exchange | 382 (141,78) | 441 (161,88) | 373 (164,91) |

**Table S3. Pearson correlation of genotype-averaged traits across three seasons.**

| **Trait** | **2021&2022** | **2021&2023** | **2022&2023** | **Mean correlation** |
| --- | --- | --- | --- | --- |
| SLA | 0.473 | 0.45 | 0.361 | 0.428 |
| %C | -0.112 | 0.196 | 0.037 | 0.04 |
| %N | 0.551 | 0.441 | 0.565 | 0.519 |
| C:N | 0.568 | 0.443 | 0.571 | 0.527 |
| d13C | 0.543 | 0.555 | 0.465 | 0.521 |
| d15N | 0.245 | 0.092 | 0.283 | 0.207 |
| Vpmax | 0.212 | 0.392 | 0.35 | 0.318 |
| Vmax | 0.558 | 0.474 | 0.374 | 0.468 |
| Photosynthetic rate (A) | 0.463 | 0.416 | 0.393 | 0.424 |
| Stomatal conductance (Gsw) | 0.488 | 0.29 | 0.349 | 0.375 |
| iWUE (A/gsw) | 0.264 | 0.219 | 0.245 | 0.243 |
| Stomatal limitation | 0.566 | 0.329 | 0.556 | 0.484 |
| NPQ induction amplitude | 0.537 | 0.59 | 0.511 | 0.546 |
| NPQ induction rate | 0.423 | 0.568 | 0.344 | 0.445 |
| NPQ induction slope | 0.526 | 0.549 | 0.419 | 0.498 |
| NPQ relaxation amplitude | 0.604 | 0.635 | 0.55 | 0.596 |
| NPQ relaxation rate | 0.582 | 0.551 | 0.454 | 0.529 |
| NPQ relaxation offset | 0.32 | 0.301 | 0.356 | 0.326 |
| Maximum NPQ | 0.585 | 0.607 | 0.535 | 0.576 |
| Final NPQ | 0.306 | 0.37 | 0.28 | 0.319 |
| ΦPSII recovery amplitude | 0.634 | 0.658 | 0.619 | 0.637 |
| ΦPSII recovery rate | 0.599 | 0.552 | 0.452 | 0.534 |
| ΦPSII recovery offset | 0.6 | 0.616 | 0.654 | 0.623 |
| Final ΦPSII | 0.429 | 0.482 | 0.441 | 0.451 |
| Fv/Fm | 0.475 | 0.521 | 0.487 | 0.495 |

**Table S4. Statistics of estimated optimal NoC for each repetition of 5-fold CV using MSE-based method (**$\boldsymbol{NoC}_{\boldsymbol{MSE(opt)}}$**) and final NoC across 20 repetitions (**$\boldsymbol{NoC}_{\boldsymbol{MSE}}$**) using the dataset from season 2021 as calibration data.** The median R^2^ was computed across all 20 × 5 test folds using either ${NoC}_{MSE(opt)}$ ($R_{{NoC}_{MSE}}^{2}$) or ${NoC}_{MSE}$ ($R_{{NoC}_{MSE(opt)}}^{2}$).

| **Trait** | $\mathrm{NoC}_{\mathrm{MSE}}$ | $R_{\mathrm{NoC}_{\mathrm{MSE}}}^{2}$ | $SD R_{\mathrm{NoC}_{\mathrm{MSE}}}^{2}$ | $SD \mathrm{NoC}_{MSE(opt)}$ | $R_{\mathrm{NoC}_{MSE(opt)}}^{2}$ | ${SD R}_{\mathrm{NoC}_{MSE(opt)}}^{2}$ |
| --- | --- | --- | --- | --- | --- | --- |
| SLA | 23 | 0.76 | 0.025 | 2 | 0.76 | 0.025 |
| %C | 1 | -0.01 | 0.033 | 0 | -0.01 | 0.033 |
| %N | 7 | 0.50 | 0.078 | 0 | 0.50 | 0.078 |
| C/N | 7 | 0.55 | 0.082 | 3 | 0.57 | 0.080 |
| d13C | 7 | 0.12 | 0.061 | 4 | 0.12 | 0.058 |
| d15N | 17 | 0.06 | 0.090 | 7 | 0.06 | 0.088 |
| V_pmax_ | 4 | 0.12 | 0.080 | 3 | 0.13 | 0.088 |
| V_max_ | 18 | 0.62 | 0.082 | 3 | 0.63 | 0.085 |
| A | 14 | 0.38 | 0.101 | 2 | 0.41 | 0.102 |
| Gsw | 9 | 0.21 | 0.093 | 4 | 0.23 | 0.098 |
| iWUE | 5 | 0.01 | 0.089 | 2 | -0.01 | 0.078 |
| SL | 4 | 0.01 | 0.063 | 2 | 0.01 | 0.062 |
| NPQ ind. amplitude | 27 | 0.30 | 0.049 | 1 | 0.31 | 0.048 |
| NPQ ind. rate | 14 | 0.05 | 0.029 | 5 | 0.04 | 0.033 |
| NPQ ind. slope | 24 | 0.11 | 0.050 | 3 | 0.09 | 0.051 |
| NPQ rel. amplitude | 27 | 0.30 | 0.050 | 1 | 0.31 | 0.050 |
| NPQ rel. rate | 23 | 0.23 | 0.043 | 3 | 0.24 | 0.043 |
| NPQ rel. offset | 6 | 0.09 | 0.030 | 1 | 0.08 | 0.030 |
| ΦPSII rec. amplitude | 29 | 0.30 | 0.047 | 1 | 0.30 | 0.046 |
| ΦPSII rec. rate | 24 | 0.25 | 0.043 | 1 | 0.26 | 0.043 |
| ΦPSII rec. offset | 27 | 0.50 | 0.042 | 3 | 0.50 | 0.044 |
| Final ΦPSII | 16 | 0.29 | 0.050 | 2 | 0.30 | 0.050 |
| Fv/Fm | 25 | 0.34 | 0.046 | 2 | 0.33 | 0.048 |
| MaxNPQ | 29 | 0.34 | 0.057 | 1 | 0.35 | 0.055 |
| Final NPQ | 8 | 0.10 | 0.032 | 2 | 0.10 | 0.033 |

**Table S5. Statistics of estimated optimal NoC for 20 repetitions of 5-fold CV using PRESS-based (**$\boldsymbol{NoC}_{\boldsymbol{PRESS}}$**) using the dataset from season 2021 as calibration data.** The median R^2^ was computed across all 20 × 5 test folds using ${NoC}_{PRESS}$ ($R_{{NoC}_{PRESS}}^{2}$).

| **Trait** | $\mathrm{NoC}_{PRESS}$ | $R_{\mathrm{NoC}_{PRESS}}^{2}$ | $std R_{\mathrm{NoC}_{PRESS}}^{2}$ |
| --- | --- | --- | --- |
| SLA | 19 | 0.76 | 0.027 |
| %C | 1 | -0.01 | 0.034 |
| %N | 7 | 0.51 | 0.07 |
| C/N | 8 | 0.57 | 0.085 |
| d13C | 6 | 0.11 | 0.062 |
| d15N | 17 | 0.09 | 0.082 |
| V_pmax_ | 4 | 0.11 | 0.087 |
| V_max_ | 18 | 0.62 | 0.076 |
| A | 14 | 0.40 | 0.094 |
| Gsw | 9 | 0.22 | 0.100 |
| iWUE | 3 | 0.00 | 0.066 |
| SL | 3 | 0.00 | 0.050 |
| NPQ ind. amplitude | 27 | 0.29 | 0.051 |
| NPQ ind. rate | 13 | 0.04 | 0.028 |
| NPQ ind. slope | 20 | 0.10 | 0.050 |
| NPQ rel. amplitude | 29 | 0.30 | 0.052 |
| NPQ rel. rate | 23 | 0.23 | 0.045 |
| NPQ rel. offset | 5 | 0.09 | 0.031 |
| ΦPSII rec. amplitude | 29 | 0.31 | 0.051 |
| ΦPSII rec. rate | 24 | 0.26 | 0.047 |
| ΦPSII rec. offset | 27 | 0.50 | 0.040 |
| Final ΦPSII | 19 | 0.32 | 0.050 |
| Fv/Fm | 21 | 0.32 | 0.052 |
| MaxNPQ | 28 | 0.35 | 0.051 |
| Final NPQ | 4 | 0.08 | 0.029 |

**Table S6. Statistics of estimated optimal NoC for each repetition of 5-fold CV** $\boldsymbol{(NoC}_{\boldsymbol{MSE(opt)}}\boldsymbol{)}$ **and final NoC across 20 repetitions (**$\boldsymbol{NoC}_{\boldsymbol{MSE}}$**) using the dataset from season 2021 as calibration data, with sub-sampled HSR data as features.** The median R^2^ was computed across all 20 × 5 test folds using either ${NoC}_{MSE(opt)}$ ($R_{{NoC}_{MSE}}^{2}$) or ${NoC}_{MSE}$ ($R_{{NoC}_{MSE(opt)}}^{2}$).

| **Trait** | $\mathrm{NoC}_{\mathrm{MSE}}$ | $R_{\mathrm{NoC}_{\mathrm{MSE}}}^{2}$ | $SD R_{\mathrm{NoC}_{\mathrm{MSE}}}^{2}$ | $SD \mathrm{NoC}_{MSE(opt)}$ | $R_{\mathrm{NoC}_{MSE(opt)}}^{2}$ | ${SD R}_{\mathrm{NoC}_{MSE(opt)}}^{2}$ |
| --- | --- | --- | --- | --- | --- | --- |
| SLA | 22 | 0.76 | 0.026 | 4 | 0.76 | 0.025 |
| %C | 1 | -0.01 | 0.033 | 0 | -0.01 | 0.033 |
| %N | 7 | 0.50 | 0.078 | 1 | 0.50 | 0.077 |
| C/N | 7 | 0.56 | 0.082 | 3 | 0.57 | 0.081 |
| d13C | 9 | 0.13 | 0.063 | 4 | 0.12 | 0.058 |
| d15N | 17 | 0.06 | 0.088 | 7 | 0.06 | 0.088 |
| V_pmax_ | 4 | 0.12 | 0.08 | 3 | 0.13 | 0.089 |
| V_max_ | 18 | 0.62 | 0.082 | 3 | 0.62 | 0.085 |
| A | 14 | 0.38 | 0.101 | 2 | 0.40 | 0.104 |
| Gsw | 9 | 0.21 | 0.094 | 3 | 0.22 | 0.096 |
| iWUE | 5 | 0.01 | 0.089 | 2 | -0.01 | 0.078 |
| SL | 4 | 0.01 | 0.063 | 2 | 0.01 | 0.061 |
| NPQ ind. amplitude | 30 | 0.31 | 0.049 | 2 | 0.30 | 0.048 |
| NPQ ind. rate | 17 | 0.05 | 0.037 | 5 | 0.04 | 0.034 |
| NPQ ind. slope | 22 | 0.10 | 0.050 | 3 | 0.10 | 0.050 |
| NPQ rel. amplitude | 29 | 0.31 | 0.050 | 1 | 0.31 | 0.050 |
| NPQ rel. rate | 27 | 0.24 | 0.044 | 3 | 0.24 | 0.046 |
| NPQ rel. offset | 6 | 0.09 | 0.030 | 1 | 0.09 | 0.030 |
| ΦPSII rec. amplitude | 29 | 0.30 | 0.045 | 2 | 0.30 | 0.045 |
| ΦPSII rec. rate | 27 | 0.27 | 0.043 | 2 | 0.27 | 0.044 |
| ΦPSII rec. offset | 33 | 0.51 | 0.045 | 2 | 0.51 | 0.044 |
| Final ΦPSII | 17 | 0.30 | 0.052 | 2 | 0.30 | 0.051 |
| Fv/Fm | 21 | 0.32 | 0.048 | 2 | 0.32 | 0.048 |
| MaxNPQ | 29 | 0.34 | 0.056 | 1 | 0.34 | 0.056 |
| Final NPQ | 7 | 0.09 | 0.035 | 2 | 0.10 | 0.033 |

**Table S7. Variance component estimates (percentage of total variance) from linear mixed-effects models for all traits across three data aggregation levels.** Variance components are Year, Accession, Accession-by-Year, Accession-by-Plot, and Residual.

Due to the size of table, the full results are provided as uploaded Excel file.

**Table S8. Variance component estimates (percentage of total variance) from linear mixed-effects models using plot-averaged data across the three seasons.** For each variance component (Year, Accession, Accession-by-Year, and Residual), three columns are provided: (i) estimates from the baseline model without environmental covariates (Model 1); (ii) estimates from a model including mean temperature and PAR from the day prior to measurement as fixed effects (Model 2); and (iii) the difference between the two models.

Due to the size of table, the full results are provided as uploaded Excel file
